# Does schistosome infection affect behavior through the gut-brain axis?

**DOI:** 10.1371/journal.pntd.0013088

**Published:** 2025-06-12

**Authors:** Leigh Combrink, Johannie M. Spaan, Alexis Perret, Thomas Maehara, Britney Hyun, Dana Parker, Jennifer L. Johns, Michael S. Blouin, Kathy Magnusson, Michelle L. Steinauer

**Affiliations:** 1 School of Natural Resources and the Environment, University of Arizona, Tucson, Arizona, United States of America; 2 Department of Biomedical Sciences, Western University of Health Sciences, Lebanon, Oregon, United States of America; 3 Ecole Nationale Veterinaire d’Alfort, Maisons-Alfort, France; 4 Department of Integrative Biology, Oregon State University, Corvallis, Oregon, United States of America; 5 Department of Biomedical Sciences, College of Veterinary Medicine, Oregon State University, Corvallis, Oregon, United States of America; 6 Linus Pauling Institute, Oregon State University, Corvallis, Oregon, United States of America; Medical University of Vienna, AUSTRIA

## Abstract

Parasitic helminths infect over 2 billion people, primarily those living in poverty. Helminth infections typically establish in early childhood and persist through critical periods of growth and development, leading to cognitive deficits and/or behavioral changes. These deficits could result from the helminths themselves or due to dysbiosis of the gut microbiota and its influence on the gut-brain axis. Using two cohorts of 3-week-old female mice, we measured levels of anxiety, fear, compulsion, spatial learning, and spatial memory, between schistosome-infected and sham-exposed mice. Additionally, we compared their fecal microbiomes using 16S rRNA gene sequencing at two time points during the chronic stage of infection. Schistosome-infected mice showed higher levels of anxiety in the open field test, reduced spatial learning in the Morris water maze task, and enhanced memory retention in the novel object task. All mice performed equally on the marble bury task. Each cohort started with unique microbiota which showed marked changes in the beta diversity of their microbiota after exposure. In both cohorts, at 7- weeks post exposure, infected mice had more *Alistipes* sp. and *Bacteroides thetaiotaomicron* and less *Turicibacter* sp. and *Ligilactobacillus* sp. than uninfected mice. At 10 weeks, infected mice had more *Alistipes* sp. and fewer *Muribaculaceae* sp. Interestingly, taxon shifts in infected mice were those typically associated with protective effects on liver disease and IL-10 gut conditions, suggesting a possible protective role of the shifted microbiome. Our analyses did not indicate associations between behavioral measures and microbiome composition; however, this could be due to the strong impact of infection on the microbiome composition. Findings here uncover behavioral and cognitive impacts of schistosome infection and shed light on the complex interplay between schistosome infection, behavioral changes, and host microbiome composition, which could ultimately support future global health efforts.

## Author summary

Our research focuses on the interplay between schistosome infection, host microbiome composition, host behavior, and inflammation in a mouse model. This work aimed to understand the potential effects of schistosome-induced microbiome disruption on childhood cognitive development as schistosome infection is a common early childhood disease in many under-resourced areas. Using two cohorts of mice, we ran well-established behavioral tests looking at anxiety, fear, compulsion, spatial learning, and memory, comparing results between schistosome-infected and uninfected mice. The marked taxon shifts in the fecal microbiota communities during the chronic stages of infection were those typically associated with protective effects on liver disease and inflammatory gut conditions, suggesting a possible protective role of the shifted microbiome. This work uncovers behavioral and cognitive impacts of schistosome infection and fits well within the scope of exploring microbiome interactions towards supporting future global health efforts.

## 1. Introduction

Parasitic helminths infect over 2 billion people, disproportionately affecting children, depriving them of their health and trapping them into a cycle of poverty [1–3]. Helminth infections are long-lasting, and reinfection is common, thus they are marked by chronic inflammation and accumulating tissue damage. These long-term effects can be difficult to measure and assess, particularly in a landscape with low access to health care, lack of diagnostic tools, and significant co-morbidities. Furthermore, helminth infections typically establish in early childhood, and persist through critical periods of growth and development, such as neurologic development of the brain [4]. Therefore, helminth infections have the potential to result in neurologic and cognitive deficits that are not only difficult to quantify, but could have lifelong effects [5,6]. While these deficits could result directly from the impact of the helminths themselves or from associated effects, such as malnutrition or inflammation, another factor is the impact of helminths on the gut microbiome [5,7]. Intestinal helminth infections alter gut microbiome composition [8,9] and could affect the bidirectional communication between the gut and the central nervous system, known as the gut-brain axis [10–12], thereby affecting host cognition and behavior [13–15]. These observations, that the composition of the gut microbiota influences cognitive function and that helminths can alter the gut microbiome, have led to the hypothesis that this three-way interaction could exacerbate the cognitive deficits seen in helminth-infected children [5,16].

Schistosomes are chronic inflammatory blood flukes [17] that cause cognitive deficits and behavior change [18]. In endemic areas, schistosomes infect children shortly after birth [6,19,20] and continue to accumulate due to the long lifespan of schistosomes within humans (5–9 years for *Schistosoma mansoni*; [21]) and repeated exposure to the infectious cercariae present in freshwater habitats. Previous studies have indicated that schistosomes impact the behavior of children, with reported deficits in school attendance, school performance, and memory, as well as increased behaviors such as hyperactivity, aggression, conduct problems, anxiety, and withdrawal [18,22–25]. However, not all studies have detected behavioral or cognitive impacts of infection, and some may be subject to bias based on their design (see [25]).

While schistosomes are blood flukes and do not inhabit the intestine, in the case of *S. mansoni*, infection directly impacts the gut as the eggs move from the mesenteric veins through the intestinal wall to be excreted with feces [17]. This leads to significant disruption of gut barrier function and the translocation of bacterial lipopolysaccharide (LPS) into the bloodstream [26,27]. Thus, the pathology caused by *S. mansoni* eggs is likely to disrupt the gut microbiome. Indeed, in one study of human subjects, *S. mansoni* infection altered the gut microbiome composition compared to healthy subjects [28], findings which have been substantiated by studies in murine models [29–31].

We investigated the links between the parasite, host microbiome and host behavior due to schistosome infection in a mouse model. Specifically we asked: 1) Do schistosomes induce systemic inflammation? 2) Does *S. mansoni* infection affect behavior? 3) Does *S. mansoni* infection affect fecal microbiome composition?, and 4) Are microbiome changes associated with behavioral changes and schistosome infection? These data will provide important baseline data and insights into aspects of host-parasite interactions that could assist with the development of future schistosome treatment options.

## 2. Materials and methods

### 2.1. Ethics statement

Project approval of the IACUC received under the relevant bodies: Oregon State University (ACUP#: 4926) and Western University of Health Sciences (IACUC #: R18IACUC010).

### 2.2. Experimental overview

**Fig 1:**
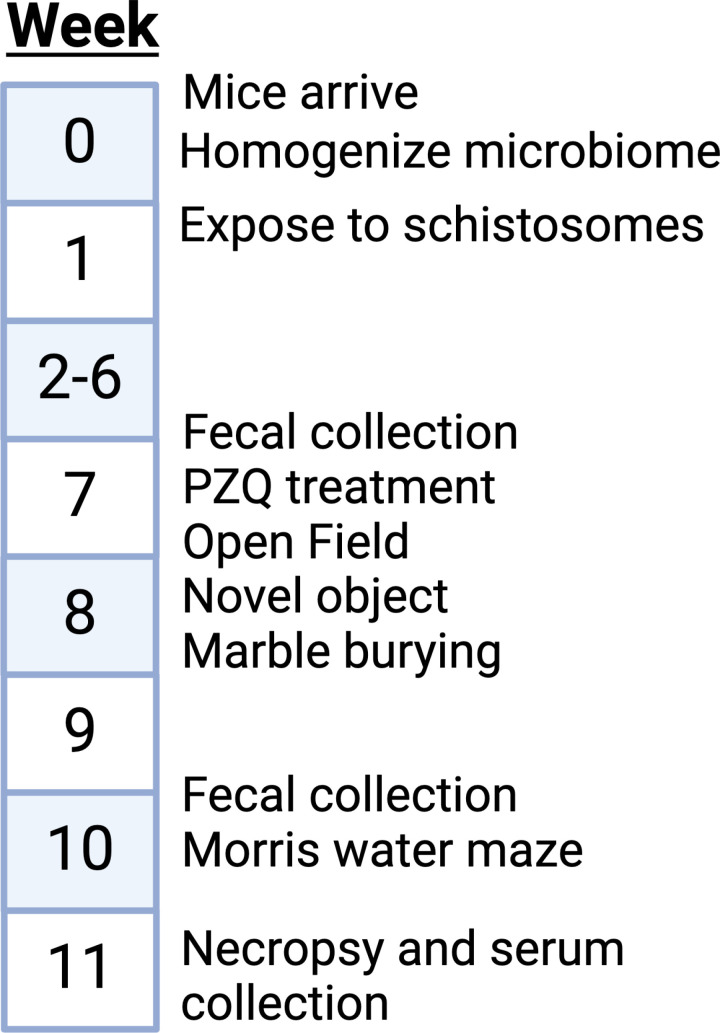
Timeline of experimental design. Created in BioRender. Steinauer, M. (2025) https://BioRender.com/ejxom55.

A timeline of the experimental overview is diagramed in Fig 1 and sample sizes per analysis are given in Table 1. Two groups of 3-week-old female C57BL/6J strain mice (group 1 and 2) arrived from The Jackson Laboratory (Bar Harbor, Maine, USA, https://www.jax.org) one week apart (N = 74). Arrival was staggered so that all behavioral analyses could be completed for each cohort in appropriate intervals. To facilitate homogenization of the microbiome between mouse groups, every other day for the first week of housing, bedding and fecal material was collected with sterile scoop and sterile gloves from each of the cages, mixed in a sterile container, and redistributed into the cages. Prior to arrival of the group 2 mice, the bedding from group 1 was sterilely collected, mixed, and distributed into the cages in which the group 2 mice were housed. The bedding from group 2 mice was mixed and redistributed three times per week as described for group 1. Mice were housed at Oregon State Universities Laboratory Animal Resource Center, 4 per cage, with standard 12/12-h light/dark cycle. They were provided with standard chow and water *ad libitum*, and given wheels for enrichment. The Oregon State University Institutional Animal Use and Care Committee reviewed and approved all procedures in this study under ACUP: R18IACUC010.

**Table 1:** Sample sizes available for each dataset across group, timing, and infection status (Inf. = Infected, Uninf. = Uninfected).

Datasets	Group 1				Group 2			
	Week 7		Week 10		Week 7		Week 10	
	Inf.	Uninf.	Inf.	Uninf.	Inf.	Uninf.	Inf.	Uninf.
Microbiome dataset	22	18	14	10	19	15	12	8
Systemic inflammation dataset	–	–	10	8	–	–	9	7
Behavioral dataset (Marble bury and Open field)	22	18	–	–	19	13	–	–
Behavioral dataset (Novel object)	20	17			18	13		
Morris water maze dataset (Mice given PZQ removed)	–	–	11	8	–	–	12	6

At four weeks of age, group 1 (n = 40) and group 2 (n = 34) cohorts were arbitrarily subdivided into control (group 1: n = 18; group 2: n = 15) and experimental groups (group 1: n = 22; group 2: n = 19) (Table A in S1 Text). The experimental mice were exposed to schistosome cercariae (NMRI line, BRI Biomedical Institute) shed from infected m-line *Biomphalaria glabrata* snails kept at Oregon State University. Infection doses varied between 50-, 75-, or 100-cercariae, with the intent to obtain a range of final worm burdens. Cercariae were pipetted into 6 cm sterile glass finger bowls containing sterile water (1 cm deep). Mice were kept in the finger bowls for 30 minutes and then returned to their cages. Control mice were sham exposed by placing them in finger bowls of water with cercariae filtered out using a 0.45 micrometer filter.

At 7 and 10 weeks post-exposure, a fecal sample was collected from each individual mouse for microbiome profiling by placing individual mice into sterile glass bowls (6 cm across) until they defecated. The fecal pellet was placed into a sterile cryovial and immediately flash-frozen in liquid nitrogen, before being stored at -80^o^C until processing. After fecal collection at 7 weeks post-exposure, the mice that were exposed to a dose of 100 parasites were given a sub-curative dose of praziquantel at 250mg/kg by oral gavage to reduce worm burdens and prevent severe illness. A subset of the control mice (n = 15) were also given praziquantel to provide a treatment control group. At 7–11 weeks post infection, all groups of mice underwent behavioral testing, including marble burying, open field, novel object and the Morris water maze as described below. Following completion of all behavioral tests, mice were euthanized.

To collect serum, blood was collected from the heart via syringe and allowed to clot in a microcentrifuge tube, which was then centrifuged at 1,000–2,000 x g for 10 minutes in a refrigerated centrifuge. A perfusion was performed on the mouse to remove the worms from the blood vessels to confirm infection status and assess worm burden. Mice that were exposed, but had no liver pathology and no worms were recovered, were classified as uninfected. We analyzed infection in two ways - classifying mice as infected or uninfected and by using the total worm burden (measured as total paired male and female worms). For statistical analysis, a Spearman’s rank test was used to show the strong correlation between the male/female paired variable and total number of worms. To determine whether worm burden were different between treatment doses (50-, 75-, or 100-cercariae), a Kruskal-Wallis rank sum test followed by Dunn’s test with Benjamini-Hochberg p-value adjustment if significant was used.

We used a multiplex immunoassay to quantify seven cytokines (IFN-γ, IL-1β, TNF-α,IL-4, IL-10, IL-17A, IL-23 cytokines (Procartaplex 7 plex, Life Technologies Corporation, New York, USA) in the serum samples collected at necropsy for a subset of mice (Table 1). For statistical analysis, to determine the effect of group and infection status on each cytokine, a Wilcoxon rank sum test with continuity correction was used, due to non-normal data. A Spearman’s rank correlation test was used to determine whether there was an association between worm burden and cytokines. The murine immune response to *Schistosoma* infection was reviewed by [32].

### 2.3. Behavioral tests

#### 2.3.1. Open field.

The open field test is commonly used in rodent models and is indicative of anxiety-like behaviors [33]. This task was performed during habituation of the mice to plastic box arenas (30.5 cm wide x 45.7 cm long x 25.4 cm high) for the novel object recognition task using 5-minute assessments. The trial was tracked and analyzed with the use of the SMART tracking system (San Diego Instruments, San Diego). Two zones were defined: the outer boundary which was 10 cm from each wall (zone 1), and the center (zone 2). The following metrics were recorded: i) the total distance traveled (%) with greater distance being indicative of healthy/exploratory behavior, ii) the permanence time spent in the outer boundary (%, total time the subject was moving within the outer boundary) with more time spent in the outer boundary being indicative of increased anxiety/fear, iii) the number of entries into the center with lower number of entrance into the center being indicative of increased anxiety/fear, and iv) the latency time before entering the center with increased time before entering the center being indicative of fear.

For statistical analysis, linear regression models (LMs), due to normally distributed data (Shapiro-Wilk’s test for normality and visual assessments) were used to determine the effect of schistosome infection on the total distance traveled and percentage time spent in the outer boundary. Generalized linear models (GLMs) with a negative binomial distribution, due to overdispersed count data and skewed distributions, were used to determine the effect of schistosome infection on the number of entries into the center, and latency time before entering the center. Group was included in each model to account for group differences.

#### 2.3.2. Novel object task.

The novel object task is used to evaluate memory based on recognition of novel objects [34,35]. Our procedure was modified from [36]. Prior to all behavioral assessments, mice were acclimated to the testing room in their home cages for at least 30 minutes, and the testing order of mice was randomized. Three habituation sessions, each on a different day, were performed for 5 minutes. After the final habituation trial and at least a 5-minute break, a familiarization session was performed with two identical objects placed in the arena. After a 1-hour break, a novel object replaced one of the identical objects for the novel object assessment. In the final assessment, which occurred 24 hours later, the novel object was replaced with a new novel object. Between assessments, objects and arenas were cleaned with 70% ethanol. Each session lasted 5 minutes and the exploration time at each object was obtained by manual timing. The percent of exploration time for each object was expressed as a percent of the total exploration time. A discrimination index was calculated as the time spent exploring the novel object minus the time exploring the familiar object divided by the total exploration time. The expectation is that if the mice remembered the familiar object after a 1 hour or 24 hour break, it will spend more time exploring the novel object.

For statistical analysis, to determine whether there were any differences in the discrimination index of schistosome infected and uninfected mice during the familiarization trial or at the 60 minute and 24 hours memory retention trials, Wilcoxon rank sum tests were used due to non-normal data as well as the presence of negative values. Mouse groups 1 and 2 were combined for this analysis because Wilcoxon rank sum test analysis indicated that there were no group differences in the metrics (Wilcoxon rank sum test, familiarization trial: *W* = 620.5, *P* = 0.8874; 60 minutes: *W* = 532, *P* = 0.4954; 24 hours: *W* = 585.5, *P* = 0.7953).

#### 2.3.3. Marble burying task.

The marble burying test is often used in rodent models to screen pharmaceuticals for treatment of compulsivity or anxiety disorders [37–40]. Marble burying cages were standard mouse microisolator cages with 10 cm Beta Chip wood shavings (Northeastern Products Corp., Warrensburg, New York, USA, http://nep-co.com) pressed and flattened and 15 glass marbles that were placed in a 3 x 5 grid. Trials lasted 30 min and the number of marbles buried more than 50% were counted as well as the number fully buried.

For statistical analysis, GLMs with negative binomial distribution due to overdispersed count data were used to determine the effect of schistosome infection on the number of marbles buried greater than 50%. Group was included in each model to account for group differences. No violation of model assumptions occurred. Very few marbles were fully buried so this measurement was not analyzed.

#### 2.3.4. Morris water maze task.

The Morris water maze test [41] for spatial learning and memory was performed as described previously with some modifications [13,42,43]. This procedure tests the ability of the mouse to learn to use spatial cues to navigate from random start points to a submerged platform in a water tank [44]. The procedure consists of repeated training “place trials” to assess spatial learning and “probe trials” to assess memory. Prior to the test, mice were acclimated to the tank for 2 days. On each day, each mouse would swim for 60 seconds in the tank without the platform or spatial cues and then trained to remain on the platform above water for 30 seconds. Assessments occurred over 3 days and included a series of intermixed place and probe trials with 5-minute cage rests in between place trials, and 90 minutes prior to probe trials (as diagrammed in Fig A in S1 Text). For all trials, the entry point of the mouse was randomized and the platform, if present, always remained in the same place. For place trials, mice were allowed to search for the platform for 60 seconds, and if they did not find the platform in that time, they were guided to the platform. Mice remained on the platform for 30 seconds and then returned to their home cages. In the probe trials, the platform was removed, and the animal was allowed to search for it for 30 seconds.

The corrected cumulative distance was calculated for each place trial, based on the animal’s cumulative distance to the platform, corrected for start position and average swim speed. The average corrected cumulative distance was calculated by taking the average for every 4 consecutive place trials and denoted as E1-E6 (Fig A in S1 Text) and used in the statistical model. For probe trials, the corrected average proximity from the platform was recorded for each mouse and recorded as Probe 0–3 (PR0-PR3). For statistical analysis, to assess the effect of schistosome infection on spatial learning (averaged place trials E1-E6) of mice, an ANOVA with type III sum of squares and a linear mixed effects model (LMM) were used to account for the random effect of repeated measures of individual mice [45]. Similarly, an ANOVA and LMM was used to determine the effect of schistosome infection on memory of mice (probe trials PR0–3). The average corrected cumulative distance for place trials were log transformed due to violation of the normality assumption. No other violation of model assumptions occurred. In both cases (place and probe trials) we were interested in the interaction term between infection status and place/probe trials.

All statistical analyses were performed in R version 4.2.2 [46], including packages ggplot2 [47], patchwork [48], MASS [49], car [50], vegan [51], dplyr [52], scales [53], grid, reshape2 [54], phyloseq [55], magrittr [56], and geosphere [57].

### 2.4. Effect of *S. mansoni* infection on the fecal microbiome composition

The fecal pellets were homogenized in Zymo fecal collection tubes containing DNA/RNA shield and silica beads using a vortex at max speed for 60s. Samples were shipped to Zymo Research and processed with the ZymoBIOMICS services (Zymo Research, Irvine, CA) for DNA extraction, PCR amplification and 16S rRNA gene sequencing of the V3-V4 region using primers 341F and 805R. The final library was sequenced on IlluminaMiSeq with a v3 reagent kit (600 cycles) and >10% PhiX spike-in (Zymo Research, Irvine, CA).

We followed the DADA2 pipeline [58] to identify sequence variants (ASVs), trim adapter sequences and remove chimeras, with sequences being processed using the following trimming parameters: truncLen = c(260, 220), trimLeft = c(17,21), maxN = 0, maxEE = c(2,2), truncQ = 2, rm.phix = TRUE. We used the default parameters for estimation of error rates using learnErrors() and removed chimeras using removeBimeraDenovo(method = “consensus”). Samples were rarified to the minimum sequencing depth of 7265 reads per sample. We used the SILVA v.138 databases for taxonomy and species assignments and constructed a phylogenetic tree by aligning sequences using mothur [59] and FastTree (v.2.1) for nucleotides. In the analyses we report the lowest taxonomic unit generated in the analysis with the caveat that 16S rRNA gene sequences do not always have the resolution to discriminate species or lower taxonomic groups [60].

Despite our efforts to homogenize gut microbiome composition between mouse groups, initial analysis of microbiome composition indicated that majority of the variance (PC1 axis 41.69%, Fig B in S1 Text) was owing to group membership. This made it essential to analyze group datasets independently. Microbiome richness was calculated as the number of unique ASVs per samples using the function estimate_richness in the R package phyloseq (v.1.40.0). Shannon diversity (sensitive to rare taxa) and evenness were also calculated using the phyloseq R package. We used Wilcoxon rank-sum tests to compare alpha diversity metrics between infected and uninfected mice for both group and time comparisons.

Beta diversity was assessed at 7- and 10-weeks post exposure using Bray-Curtis distance calculations of abundance, principle co-ordinates analysis and data visualizations (phyloseq package). PERMANOVAs (adonis2 function in the vegan package v.2.6-2) were used to assess associations of microbiome composition between infected and uninfected mice. Sample sizes differed between time points because mice that received praziquantel treatment after the 7-week time point, were removed from the 10-week analysis, due to the potential effects that the treatment could have on the microbiome. Furthermore, in some of these cases, the praziquantel treatment cleared infection, making classification into infection groups based on infection, ambiguous (Table 1). Full data (including the mice that received PZQ treatment) are given in Figs C and D in S1 Text and the effect of praziquantel administration at the 10-week time point between control groups are given in Fig E in S1 Text.

To identify taxa that differed significantly between infected and uninfected mice, we used Linear Discriminant Analysis Effect Size (LEfSe) [61], which conducts non-parametric tests between chosen groups to identify significant taxa and the effect size associated with each taxa identified (standard/default settings for LEfSe’s were used). Given the strong signal of the genus *Alistipes* and *Bacteroides thetaiotaomicron* as markers of infection, we asked if the relative abundance of these taxa increase over time, from week 7–10, in infected mice, and if relative abundance increases with worm burden. Spearman’s rank correlations were used to determine whether the relative abundance of *Alistipes*/*B. thetaiotaomicron* correlates with worm burden across both groups at all time points. To determine whether there was an increase in relative abundance of *Alistipes*/*B. thetaiotaomicron* over time (from 7-10 weeks from a normal to disease state), a Wilcoxon signed rank test was used for all comparisons (across groups and infection status), due to paired data.

### 2.5. Assessing the interaction between schistosome infection and behavioral measurements on alpha and beta diversity metrics

Either ANOVA with type III sum of squares and linear regression models or generalized linear model with negative binomial distribution (depending on violation of the normality assumption) was used to assess the interaction between schistosome infection and behavioral measurements on alpha diversity metrics. To assess the interaction between schistosome infection and behavioral measurements on the microbiome composition, we performed PERMANOVAs. Given the timing of the tests (Fig 1), we used the week 7 microbiome data for the open field, novel object, and marble burying tasks, and week 10 data for the Morris water maze task. To ensure that praziquantel administration did not significantly affect the mouse behavior outcomes that matched the 7-week time point, we used a Mann-Whitney U test to compare control mice and praziquantel controls against the behavioral metrics (see Table B in S1 Text for no praziquantel effect).

## 3. Results

### 3.2. Infection and inflammation

**Fig 2:**
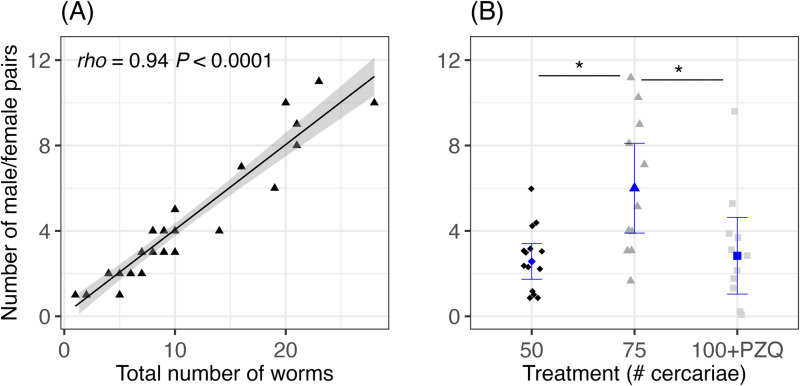
Description of the schistosome burdens in mice. (A) strong correlation between the total number of worms and the number of male/female pairs (Spearman’s rank correlation), and (B) the number of male/female pairs by treatment dose of 50 (black, diamonds, n = 14), 75 (dark gray, triangles, n = 12), or 100 (light gray, squares, n = 15) cercariae. Dose 100 received praziquantel (PZQ) treatment 7-weeks post exposure. Blue symbols and error bars represent the mean and 95% confidence intervals for each treatment category. The asterisk indicates a significant difference of *P* < 0.05 (Dunns’ test).

There was a strong correlation between the male/female paired worms and total number of worms (Spearman’s rank correlation, *rho* = 0.94, *S* = 410.22, *P* < 0.0001; Fig 2A). There were no group differences in worm burden for either total number of worms (Wilcoxon rank sum test, *W* = 81, *P* = 0.8914) or total number of male/female pairs (Wilcoxon rank sum test, *W* = 76, *P* = 0.9341). The mean worm burdens were 3 male/female pairs (±SD = 1) for dose 50 mice, 6 male/female pairs (±SD = 3) for dose 75 mice, and 3 male/female pairs (±SD = 3) for dose 100 mice receiving PZQ treatment (Kruskal-Wallis rank sum test, *ꭓ*^*2*^_*df=2*_ = 9.45, *P* = 0.0089, Fig 2B). Worm burden were higher in mice that were exposed to 75 cercariae compared to 50 cercariae (Dunn’s test, *P* = 0.0081) or dose 100 after PZQ treatment (Dunn’s test, *P* = 0.0069) (Fig 2B). PZQ treatment was apparently partially effective as there were no differences between mice exposed to 50 cercariae and the group exposed to 100 cercariae (Dunn’s test, *P* = 0.4673; Fig 2B).

**Fig 3:**
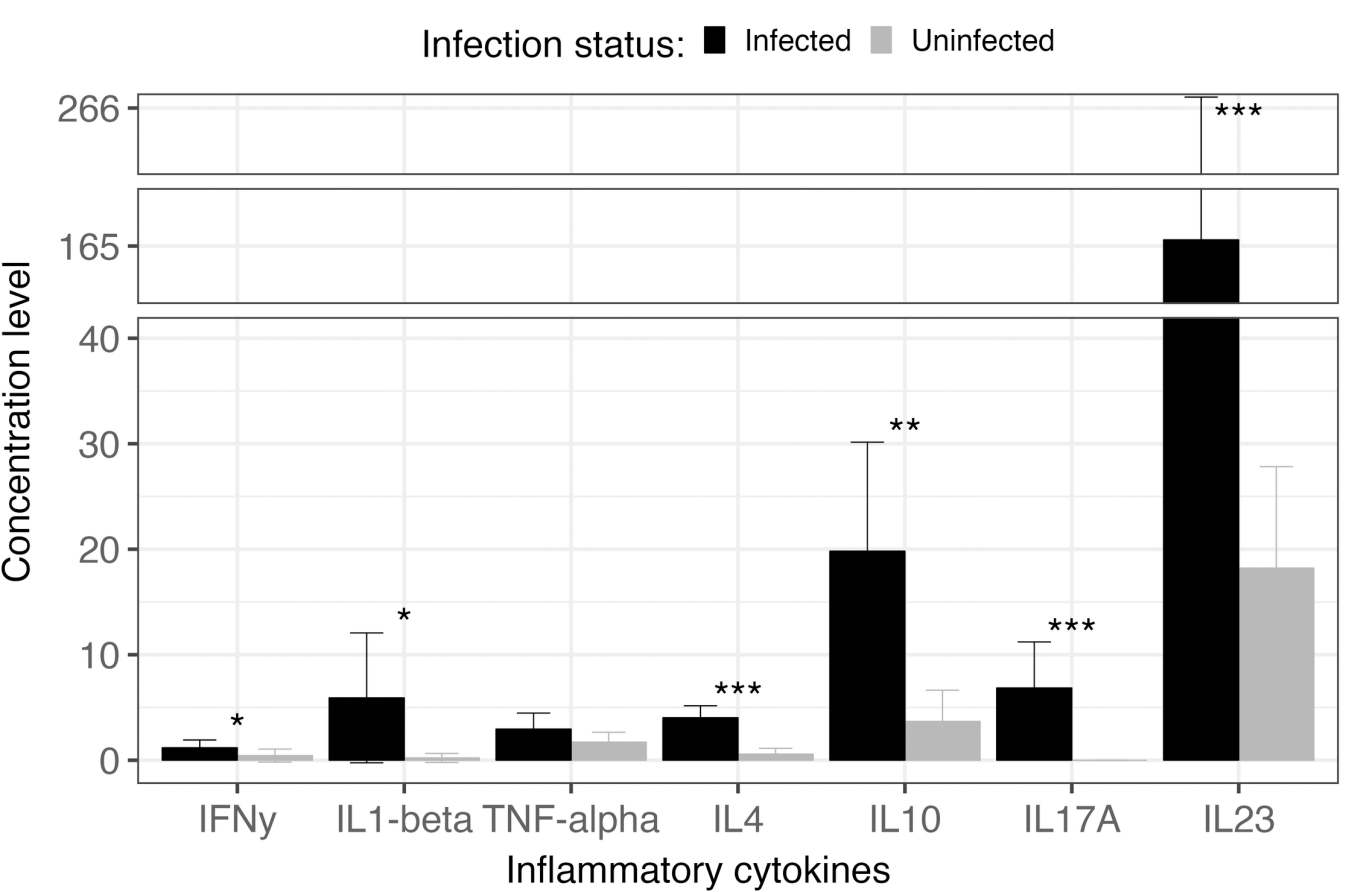
Cytokine concentrations of schistosome infected (black, n = 19) versus uninfected (gray, n = 15) mice. Error bars represent 95% confidence intervals. Asterisk indicates significant differences: * *P* < 0.05; ** *P* < 0.001; *** *P* < 0.0001.

All cytokines tested in this study, except for TNF-α, had significantly higher concentrations in schistosome-infected mice compared to uninfected mice (Fig 3). Schistosome infected mice had higher Th1 responses (Wilcoxon rank sum test; IFN-γ: *W* = 86, *P* = 0.0263; IL-1β: *W* = 91, *P* = 0.0227), Th2 responses (Wilcoxon rank sum test; IL-4: *W* = 16, *P* < 0.0001; IL-10: *W* = 49, *P* = 0.0011), and Th17 responses (Wilcoxon rank sum test; IL-17A: *W* = 52.5, *P* = 0.0003; IL-23: *W* = 30, *P* = 0.0001) compared to uninfected mice. There was no significant effect on schistosome infection on TNF-α (Th-1 response) concentrations (Wilcoxon rank sum test, *W* = 122.5, *P* = 0.4891). There was no association between worm burden and any of the cytokines.

### 3.3. The effect of schistosome infection on behavior

#### 3.3.1. Open field test.

**Fig 4:**
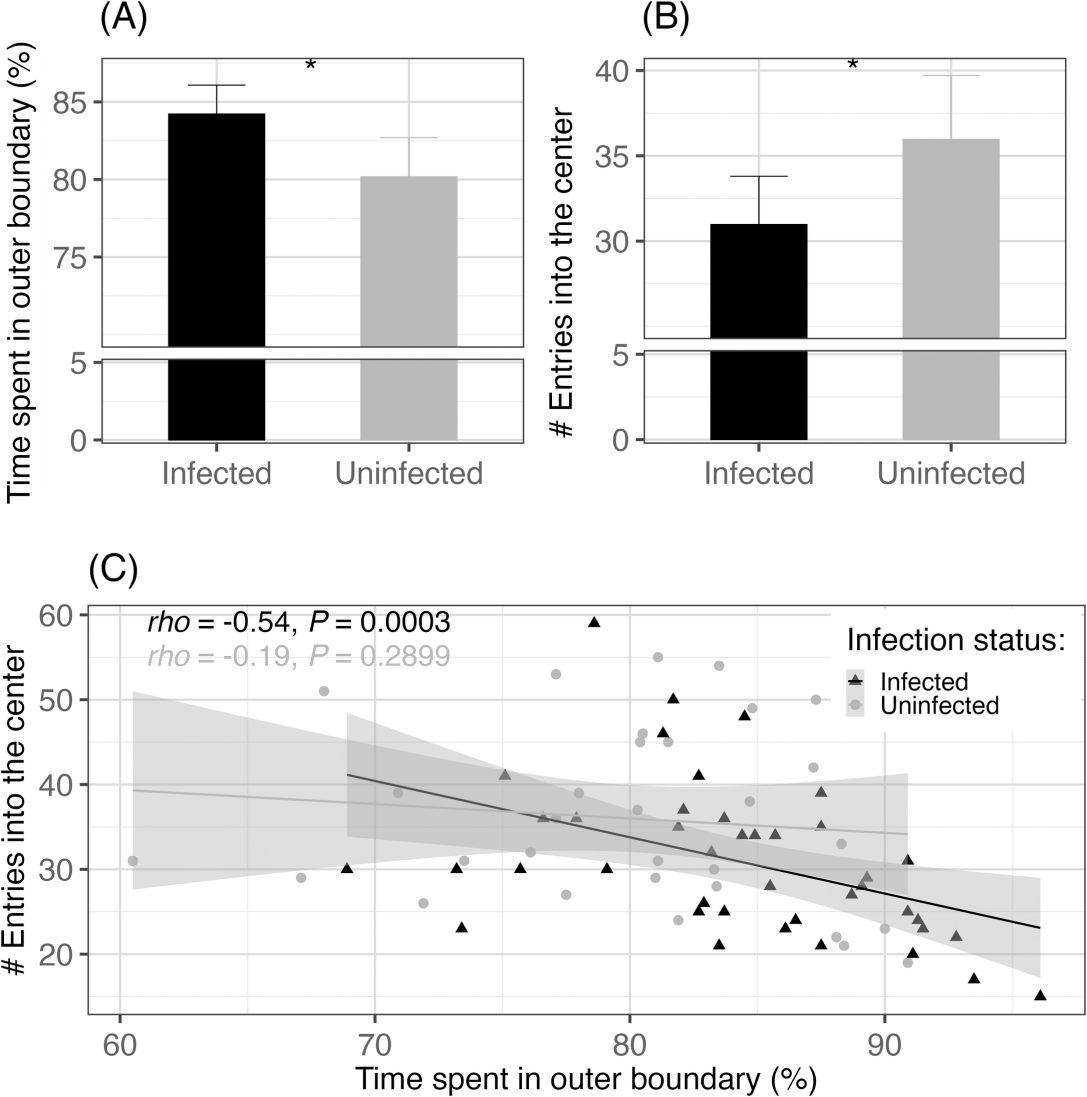
Open field test results for schistosome-infected (black, n = 41) and uninfected (gray, n = 31) mice for the (A) time spent in the outer boundary (expressed as a percentage) and (B) number of times mice entered the center. Error bars represent 95% confidence intervals. The asterisk indicates a significant difference of *P* < 0.05. (C) Correlation between the time spent in the outer boundary and the number of times mice entered the center for both schistosome infected (black, triangles) and uninfected mice (gray, circles) (Spearman’s rank correlation test).

Mice infected with schistosomes spent more time in the outer boundary, than uninfected mice, while accounting for group (LM, *β*_0_ = 79.91, *β* = 4.02, *t* = 2.6, *P* = 0.0128, Fig 4A). Mice infected with schistosomes had 14% lower odds of entering the center compared to uninfected mice (GLM, *β*_0_ = 3.57, *β* = -0.15, *z* = -2.2, *P* = 0.0274, Fig 4B), while accounting for group. Unsurprisingly, these two metrics (time spent in the outer boundary and number of times mice entered the center) were negatively correlated for infected mice (Spearman’s correlation test, *rho* = -0.54, *P* = 0.0003, Fig 4C), but less so for uninfected mice (Spearman’s rank correlation test, *rho* = -0.19, *P* = 0.2899, Fig 4C). There was no significant effect of schistosome infection on mice’s latent time before entering the center (GLM, *β*_0_ = 2.22, *β* = -0.02, *z* = -0.1, *P* = 0.9160). There was no significant effect of schistosome infection on the total distance mice traveled, while accounting for group (LM, *β*_0_ = 2417, *β* = -251.4, *t* = -1.9, *P* = 0.0661).

#### 3.3.2. Novel object task.

**Fig 5:**
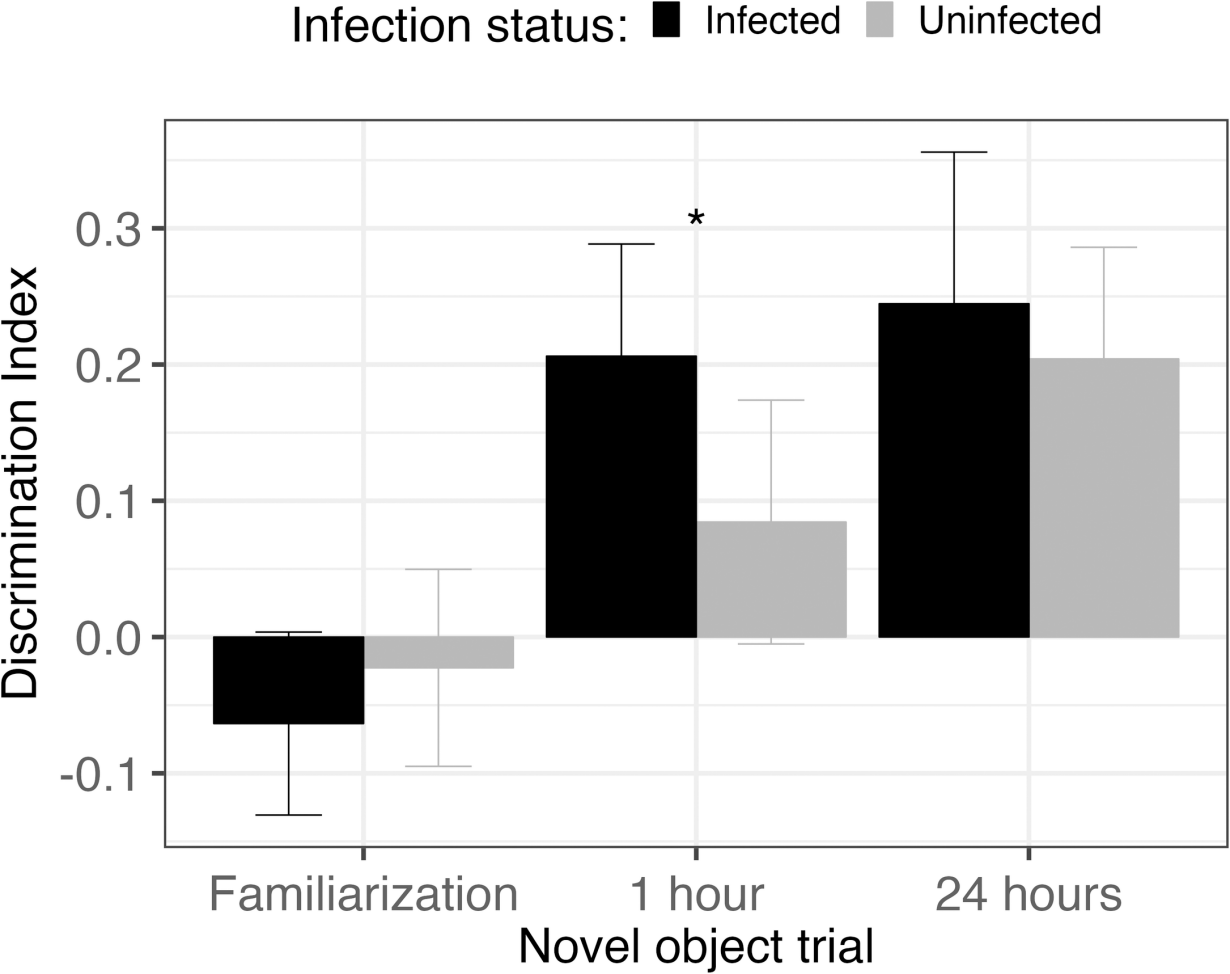
Discrimination indexes of infected (black, n = 38) and uninfected (gray, n = 30) mice from the familiarization, 1-hour and 24-hour trails. Asterisk indicates a significant difference of *P* < 0.05. Error bars represent 95% confidence intervals.

Mice infected with schistosomes apparently had greater memory retention after 1-hour than uninfected mice, but no differences were seen after 24-hours (Fig 5). During the familiarization trial of two identical objects, there were no significant differences in the discrimination index for induced preferences between infection status (Wilcoxon rank sum test, *W* = 671.5, *P* = 0.3991). After 1-hour, there was a significant difference in the discrimination indexes of infected and uninfected mice, in that infected mice spent more time exploring the novel object than uninfected mice (Wilcoxon rank sum test, *W* = 418.5, *P* = 0.0445). However, after 24-hours there was no difference in this measure (Wilcoxon rank sum test, *W* = 536, *P* = 0.4214).

#### 3.3.3. Marble bury task.

There was no significant effect of schistosome infection on the number of marbles buried, while accounting for group (GLM, *β*_0_ = 1.54, *β* = 0.29, *z* = 1.6, *P* = 0.1200).

#### 3.3.4. Morris water maze task.

**Fig 6:**
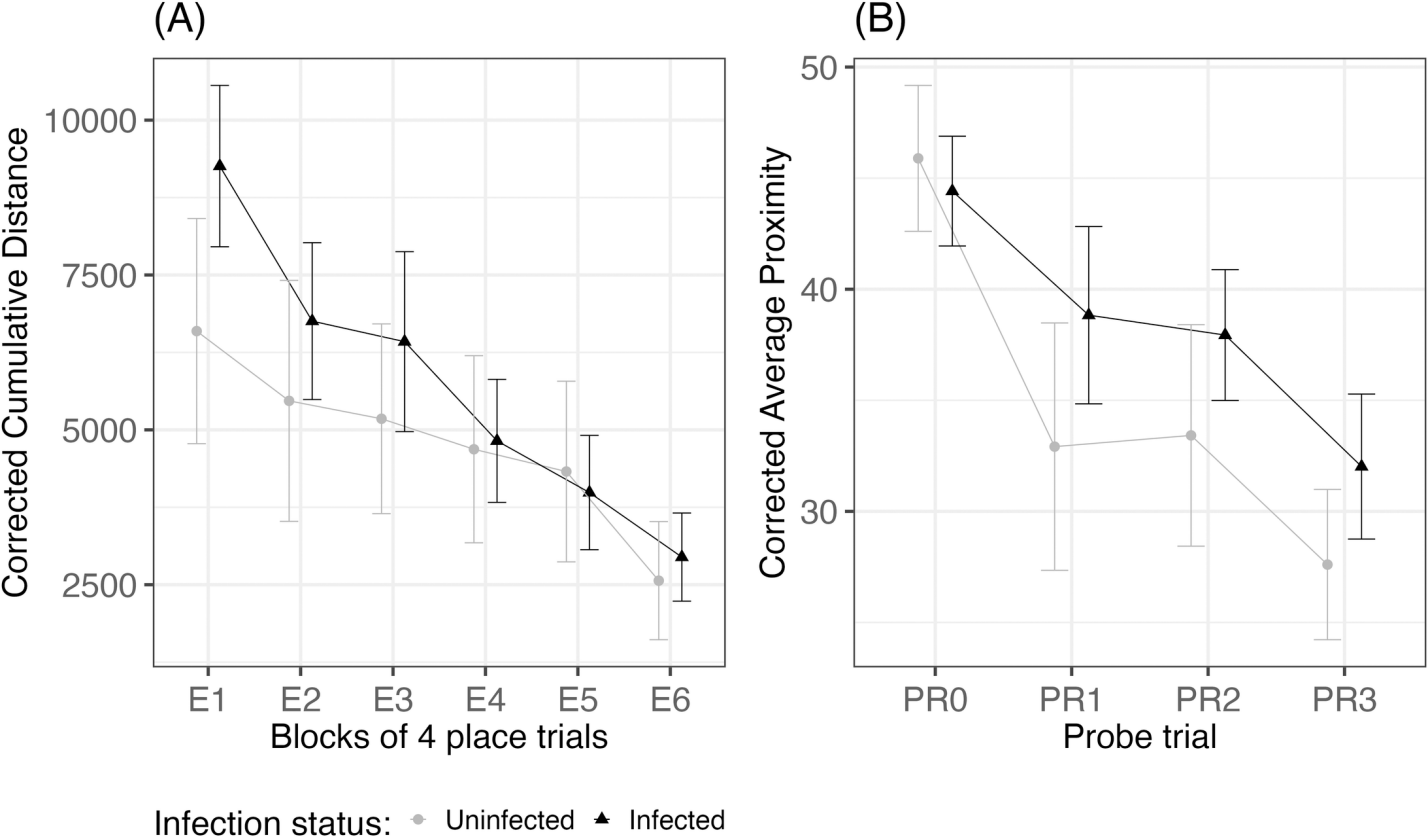
Morris water maze test results of infected (black, triangles, n = 23) and uninfected (gray, circles, n = 14) mice showing: (A) the average corrected cumulative distance across blocks of 4 place trials (E1-E6) and (B) corrected average proximity across probe trials (PR0-PR3). Error bars represent 95% confidence intervals.

The place trials indicated that infection status affected the long-term spatial learning ability of mice, as the average corrected cumulative distance (cm) was significantly lower in uninfected mice than schistosome infected mice, while accounting for group, place trials, and repeated measures of the same individual (ANOVA, *ꭓ*^*2*^_*df=1*_ = 6.2, *P* = 0.0127). There was no significant interaction in corrected cumulative distance between place trials (trials E1-E6) and schistosome infection, while accounting for group differences and repeated measures of the same individual (ANOVA, *ꭓ*^*2*^_*df=5*_* *= 7.2, *P* = 0.2050, Fig 6A). Overall, there was a significant reduction in the average corrected cumulative distance (cm) between place trial blocks E1 and E6, while accounting for infection status, group, and repeated measures of the same individual (ANOVA, *ꭓ*^*2*^_*df=5*_ = 90.3, *P* < 0.0001, Fig 6A), which indicates learning over the course of trials.

The reference memory of mice was tested through probe trials, which did not reveal any differences between infected and uninfected mice. There was no significant interaction in corrected average proximity (cm) between probe trials (trials PR0-PR3) and schistosome infection, while accounting for group differences and repeated measures of the same individual (ANOVA, *ꭓ*^*2*^_*df=3*_ = 5.7, *P* = 0.1246, Fig 6B). There was a significant reduction in corrected average proximity (cm) between probe trials PR0 and PR3, while accounting for infection status, group differences, and repeated measures of the same individual (ANOVA, *ꭓ*^*2*^_*df=3*_ = 36.3, *P* < 0.0001, Fig 6B). There was no significant difference in the corrected average proximity (cm) between schistosome infected and uninfected mice, while accounting for group differences, probe trials, and repeated measures of the same individual (ANOVA, *ꭓ*^*2*^_*df=1*_ = 0.2, *P* = 0.6565).

### 3.4. Effect of *Schistosoma mansoni* infection on the gut microbiome

**Fig 7:**
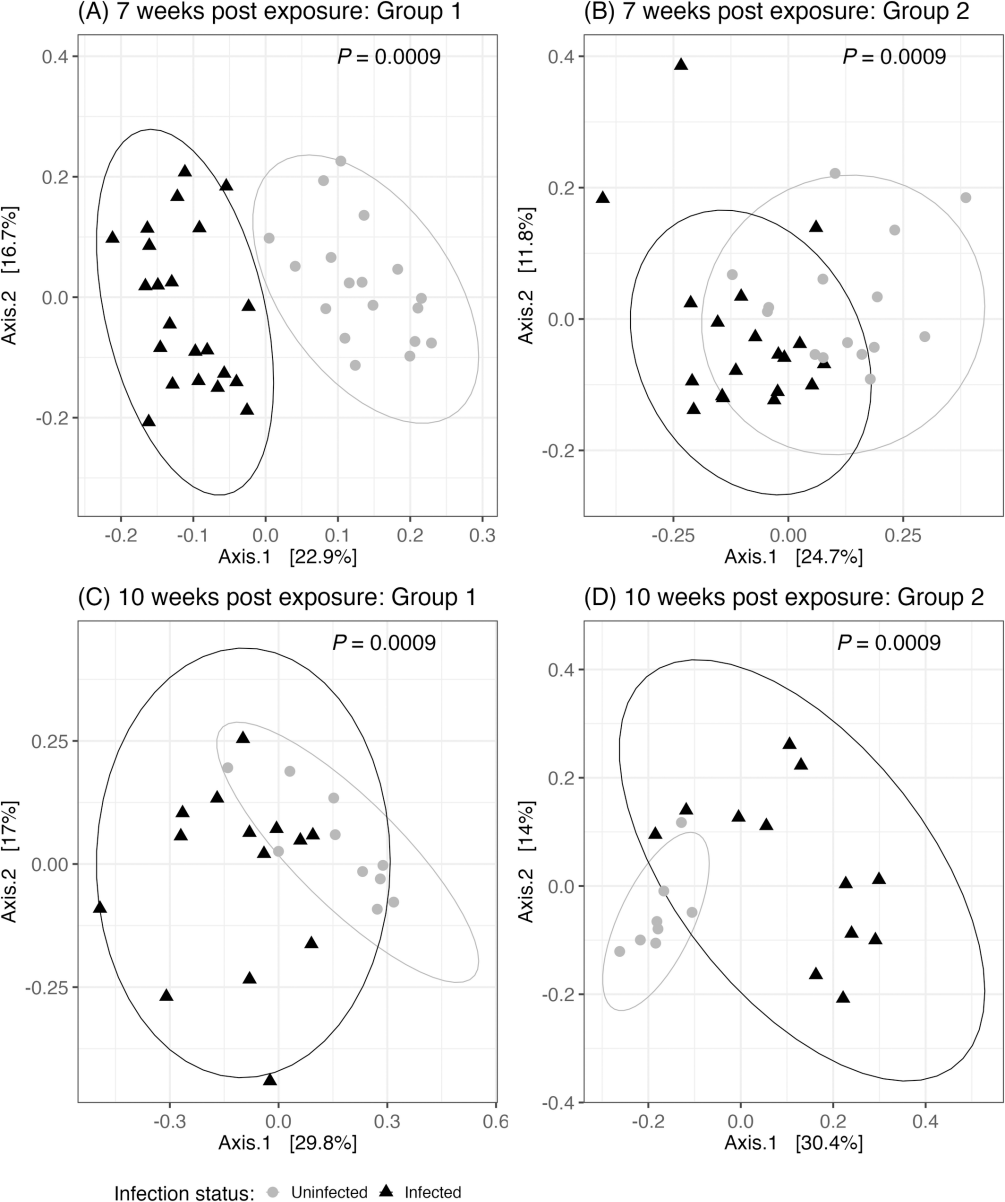
PCoA plots with ellipses comparing infected (black, triangles) and uninfected (gray, circles) mice for (A) group 1 week 7 (n = 40), (B) group 2 Week 7 (n = 34), (C) group 1 week 10 (n = 24), and (D) group 2 week 10 (n = 20). *P*-values indicate significance from PERMANOVA analysis. See Figs C and D in S1 Text for full dataset including praziquantel treated mice at 10 weeks post exposure.

Schistosome infection caused a shift in the gut microbiome composition, which differed significantly between infected and control mice for both group and time point comparisons (Fig 7). Worm burden (as measured by total number of paired worms) did not have any effect on species composition for either group 1 (PERMANOVA, *F*_1,13 _= 1.35, *P* = 0.1908) or group 2 (PERMANOVA, *F*_1,11 _= 1.64, *P* = 0.0979). Interestingly, despite finding composition changes, we found no significant effect of infection on alpha diversity at week 7 and week 10 for either group (Table C in S1 Text).

Full results of the LEfSe analyses comparing the taxa that differed between infected and control mice can be found in Figs F-I in S1 Text and a summary in Table 2. At week 7 post-infection, taxa that were more abundant in infected mice compared to controls, across both groups of mice, were the genus *Alistipes* and the species *Bacteroides thetaiotaomicron* (Table 2). Those less abundant in infected mice compared to controls included the genera *Turicibacter* and *Ligilactobacillus*. Mouse groups 1 and 2 each had unique taxa differing between infected and control mice, being mostly members of the class Clostridia and the genus *Lactobacillus* (Table 2).

**Table 2:** Summary of the taxa overrepresented in the microbiomes of schistosome infected and uninfected mice across two replicate groups of mice at 7- and 10-weeks post-exposure according to Linear Discriminant Analysis Effect Size (LEfSe) analysis. Abbreviations: G1 = Group 1; G2 = Group 2. Numbers in parentheses are the LDA scores from the LEfSe analysis.

	Infected	Uninfected
**Week 7**		
All Mice	*Alistipes* sp. (G1: 2.37; G2: 2.44)	*Turicibacter* sp. (G1: 2.56; G2: 2.47)
	*Bacteroides thetaiotaomicron* (G1: 2.15; G2: 2.11)	*Ligilactobacillus* sp. (G1: 2.44; G2: 2.26)
Group 1 only	*Clostridia* UCG-014 (2.18)	*Romboutsia ilealis* (2.02)
	Oscillospirales (2.13)	*Clostridium ss* 1 (2.62)
Group 2 only	Lachnospiraceae ASF356 (2.08)	*Lactobacillus* sp. (2.47)
	Lachnospiraceae sp. (2.30)	
**Week 10**		
All Mice	*Alistipes* sp. (G1: 2.62; G2: 2.75)	Muribaculaceae sp. (G1: 2.80; G2: 2.71)
Group 1 only	*Bacteroides thetaiotaomicron* (2.09)	*Turicibacter* sp. (2.19)
	Ruminococcaceae (2.04)	*Romboutsia ilealis* (2.00)
	*Lachnospiraceae NK4A136* (2.51)	*Clostridium ss* 1 (2.88)
	Lachnospiraceae sp. (2.99)	
Group 2 only		[Eubacterium] xylanophilum group (2.17)

At week 10 post-infection, *Alistipes* was more abundant and Muribaculaceae were less abundant in both groups of infected mice compared to controls (Table 2). As with week 7, each mouse group each had unique taxa that differed between infected and control mice, mostly members of the class Clostridia (Table 2). The species *B. thetaiotaomicron* were still more abundant and the genus *Turicibacter* were still less abundant in infected mice compared to controls, but only in group 1 (Table 2).

*Alistipes* relative abundance increased over time from week 7 to week 10 in infected mice (Wilcoxon signed rank test; Group 1: *V *= 85, *P* = 0.0419; Group 2: *V* = 71, *P* = 0.0093), however, there was no significant correlation with worm burden at 7 weeks post-exposure (Spearman’s rank correlation; Group 1: *rho* = 0.37, *S* = 288.2, *P* = 0.1972; Group 2: *rho* = 0.50, *S* = 143.5, *P* = 0.0992) or 10 weeks post-exposure (Spearman’s rank correlation; Group 1: *rho* = 0.22, *S* = 354.9, *P* = 0.4498; Group 2: *rho* = 0.48, *S* = 148.6, *P* = 0.1138). *B. thetaiotaomicron* relative abundance did not change across time points for both groups (Wilcoxon signed rank test; Group 1: *V *= 52, *P* = 0.9999; Group 2: *V *= 14, *P* = 0.0523). The relative abundance of *B. thetaiotaomicron* was positively correlated with worm burden at 7 weeks post-exposure, in group 2 mice only (Spearman’s rank correlation; Group 1: *rho* = 0.50, *S* = 227.5, *P* = 0.0686; Group 2: *rho* = 0.58, *S* = 119.2, *P* = 0.0466), and at week 10, in group 1 mice only (Spearman’s rank correlation; Group 1: *rho* = 0.56, *S* = 200.2, *P* = 0.0373; Group 2: *rho* = 0.10, *S* = 257.7, *P* = 0.7597).

### 3.5. Assessing the interaction between schistosome infection and behavioral measurements on alpha or beta diversity metrics

There was no clear association between gut microbiome and any of the behavioral measures tested for in this study. There were only few significant interactions between schistosome infections and behavioral measurements on species richness, species evenness, or Shannon index metrics with no clear pattern (Table D in S1 Text). Even less so for the interaction effect between schistosome infections and behavioral measurements on species composition (Table E in S1 Text).

## 4. Discussion

Mice infected with schistosomes showed a marked shift in the fecal microbiota communities at 7- and 10-weeks post infection. The relationship between gut microbiota and schistosome infection is likely to be multidimensional, with previous work indicating that the microbiota impacts host susceptibility to schistosomes [30,62–65], and the progression of schistosome induced pathology [66]. In this study, we investigated links between schistosome infection, microbiome composition, and host cognition/behavior, which could explain cognitive declines and behavior shifts detected in humans infected with schistosomiasis [18,22–25]. Schistosome infected mice showed higher levels of anxiety as detected by decreased entries into the middle of the arena in the open field assay. Because total distance did not differ between control and infected mice, this difference does not seem to be due to illness, depression, or lack of mobility. A previous behavioral study of schistosome infected mice indicated similar findings in that 8-week infected mice showed increased anxiety as well as increased exploration [67], though the behavioral assays differed quite significantly from those in the current study. Other non-schistosome helminths induce anxiety in rodent models, and in some cases, induced anxiety has been correlated with a shift in microbiome composition [7,68,69].

Schistosome-infected mice also showed reduced spatial learning compared to uninfected mice as measured by the place trials of the Morris water maze task. Reduced learning, memory and scholastic achievement has been reported for human children infected with schistosomes [24,25]. Furthermore, systemic inflammation (TNF-α and IL-6) together with schistosome infection has been linked to low performance in learning among school children [70]. In mice, previous studies have shown a role for the microbiome in learning and particularly that learning was enhanced in mice with an overabundance of *Lactobacillus* or upon supplementation with *Lactobacillus* or lactate, a metabolite produced by *Lactobacillus* and other lactic acid bacteria [71]. In the current study, *Lactobacillus* was more common in uninfected mice than infected mice in group 2 only; however, uninfected mice in both groups showed higher levels of *Ligilactobacillus,* which also produce lactate. Thus, a reduction in the lactic acid producing bacteria could be contributing to the lower performance of the schistosome-infected mice. Additionally, the systemic inflammation caused by the infection could be driving or exacerbating the effects on cognitive function and anxiety behaviors in mice [72].

A puzzling result of the novel object assay was the increased exploration of the novel object one hour after the familiarization trial by infected mice compared to uninfected mice. Typically, increased exploration of the novel object compared to the familiar object is interpreted as enhanced short-term memory of the familiar object [34,35]. Inequivalent total exploration between the groups potentially could explain this finding; however, in the current study, there were no differences in the total object exploratory time. Previous work found that helminth excretory-secretory products (ESPs) ameliorated obesity induced cognitive deficits in a mouse model [73]; however, it seems less likely in this case that ESPs would enhance cognition above control mice that have no cognitive deficits. On the other hand, such neuroinflammatory-reducing ESPs released by schistosomes could explain the overall lack of behavioral changes detected in this study despite microbiome dysbiosis and high levels of systemic inflammation. In other words, the immunomodulatory capabilities of helminths, that function to enable chronic infections, may provide protection for the host against neuroinflammation.

Microbiome changes were primarily detected in beta diversity measures. The genus *Alistipes* was strongly associated with infection, being overabundant compared to controls in both groups of mice, at both time points. Therefore, this group could potentially serve as a biomarker of infection. Other taxa that were overabundant in infected animals were *Bacteroides thetaiotaomicron*, and members of the Lachnospiraceae. These observations are somewhat counterintuitive to expectations based on the major pathologies of schistosomiasis, which are liver fibrosis and colitis [74]. Previous studies have shown that the gut microbiota play a role in the progression of liver fibrosis as well as intestinal inflammation and colitis; however, the patterns observed in our data was somewhat opposite. For instance, in previous studies, *Alistipes* (overabundant in infected mice) has been negatively associated with liver diseases [75–77] and have been found to suppress inflammation [78]. *Alistipes* have also been found to be protective against liver disease [79,80] and colitis [81]. Likewise, *B. thetaiotaomicron* (overabundant in infected mice in our study), ameliorates pathology of induced colitis in a mouse model by promoting anti-inflammatory Th2 and Treg cells, and suppressing proinflammatory Th1/Th17 cells [82,83]. Furthermore, supplementation with this species has been shown to reduce hepatic steatosis and restore mucosal barrier in a mouse model of non-alcoholic fatty liver disease [84] and alcoholic fatty liver disease [85]; however this benefit was not reproduced by [86]. It is possible that these counterintuitive changes in microbial communities are related to the ability of schistosomes to modulate the immunological response of the host to reduce pathologic consequences [87,88]. In fact, in a previous study of liver fibrosis of a mouse model caused by *Schistosoma japonicum* found that granulomas and fibrosis were negatively correlated with *Alistipes* and Lachnospiraceae abundance [89], supporting the idea that these taxa may be protective.

The taxa that were reduced in infected animals compared to controls included: *Ligilactobacillus, Turicibacter*, and *Muribaculaceae*. *Ligilactobacillus* is often considered a beneficial microbe and used as a probiotic (e.g., [90,91]) to enhance gut barrier function and protect against infection [92,93].

Interestingly, one previous study detected an increase in Lachnospiraceae with schistosome infection of mice [29], similar to our findings; however another showed a mixed pattern with different members of Lachnospiraceae [30]. Abundance of *Alistipes* and *Bacteroides* was also previously found to be increased in schistosome infected mice [29,31,89,94] and Muribaculaceae [31] and *Turicibacter* [29] were found to be less abundant in schistosome infected mice, consistent with our findings.

Our results showed that schistosome infected mice and their controls were similar in diversity and richness of the fecal microbiota (alpha diversity). Previous studies of *S. mansoni* infected mice have shown mixed results with either a decrease [29,30]; or no significant difference [31]. However, these studies all differed in the mouse models, parasites, and timing of samples, thus direct comparisons are challenging. Moreover, there are few general patterns in taxonomic changes resulting from schistosome infection and most are unique to the study cohort. Our experimental design that utilized two cohorts of mice from the same vendor, ordered one week apart, and housed in the same vivarium room, under the same conditions underscore this conclusion.

In summary, schistosome infection has a large impact on the fecal bacterial microbiota composition of female mice, and these changes could have both protective effects for the host as well as negative impacts on cognitive function and behavior with increased anxiety, and decreased learning. Additionally, despite previous work linking gut inflammation and reduced gut barrier integrity to neuroinflammation and behavioral changes (e.g., [13,95]), we did not find a direct interaction between behavior and microbiome composition in schistosome infected mice. It could be that they are not directly related, or that the relationship among infection, microbiota, and behavior change are too highly correlated to be teased apart with this study design.

## Supporting information

S1 Text **Fig A.** Diagram displaying place and probe trial experiment over three days. **Fig B.** PcoA analyses to show baseline differences between group 1 and group 2. **Fig C.** PcoA plots comparing infected, infected with praziquantel, control and control with praziquantel mice by week and group with the full data for the 10-week time point, including the mice that received praziquantel treatment. **Fig D.** PcoA plots comparing infected, infected with praziquantel, control and control with praziquantel mice by week and group with the full data for the 10-week time point, including the mice that received praziquantel treatment. Cage number for each individual mouse is shown. **Fig E.** PCoA plots comparisons between control mice with praziquantel and control mice at 10 weeks post exposure for both groups. **Fig F.** LEfSe analysis comparing the taxa that differed significantly between schistosome infected and uninfected mice from group 1 at 7 weeks post exposure. **Fig G.** LEfSe analysis comparing the taxa that differed significantly between schistosome infected and uninfected mice from group 2 at 7 weeks post exposure. **Fig H.** LEfSe analysis comparing the taxa that differed significantly between schistosome infected and uninfected mice from group 1 at 10 weeks post exposure. **Fig I.** LEfSe analysis comparing the taxa that differed significantly between schistosome infected and uninfected mice from group 2 at 10 weeks post exposure. **Table A.** Breakdown of sample sizes by cage, group, cercariae dose and treatment, and infection status (Yes/No). **Table B.** Comparisons between control mice and control praziquantel mice across all behavioral metrics. **Table C.** Effects of infection status on alpha diversity metrics for each group and time comparison. **Table D.** Regression outputs for the interaction between infection status and behavioral metrics on alpha diversity metrics for each group. **Table E.** PERMANOVA outputs for the interaction between infection status and behavioral metrics on beta diversity metrics (Bray-Curtis dissimilarity index) for each group.
(DOCX)
