# Supplementary material for: Does schistosome infection affect behavior through the gut-brain axis?
Source: PLoS Negl Trop Dis. 2025 Jun 12;19(6):e0013088. doi: 10.1371/journal.pntd.0013088 (PMC12184923; doi:10.1371/journal.pntd.0013088)
Supplement: S1 Text — Fig A. Diagram displaying place and probe trial experiment over three days. Fig B. PcoA analyses to show baseline differences between group 1 and group 2. Fig C. PcoA plots comparing infected, infected with praziquantel, control and control with praziquantel mice by week and group with the full data for the 10-week time point, including the mice that received praziquantel treatment. Fig D. PcoA plots comparing infected, infected with praziquantel, control and control with praziquantel mice by week and group with the full data for the 10-week time point, including the mice that received praziquantel treatment. Cage number for each individual mouse is shown. Fig E. PCoA plots comparisons between control mice with praziquantel and control mice at 10 weeks post exposure for both groups. Fig F. LEfSe analysis comparing the taxa that differed significantly between schistosome infected and uninfected mice from group 1 at 7 weeks post exposure. Fig G. LEfSe analysis comparing the taxa that differed significantly between schistosome infected and uninfected mice from group 2 at 7 weeks post exposure. Fig H. LEfSe analysis comparing the taxa that differed significantly between schistosome infected and uninfected mice from group 1 at 10 weeks post exposure. Fig I. LEfSe analysis comparing the taxa that differed significantly between schistosome infected and uninfected mice from group 2 at 10 weeks post exposure. Table A. Breakdown of sample sizes by cage, group, cercariae dose and treatment, and infection status (Yes/No). Table B. Comparisons between control mice and control praziquantel mice across all behavioral metrics. Table C. Effects of infection status on alpha diversity metrics for each group and time comparison. Table D. Regression outputs for the interaction between infection status and behavioral metrics on alpha diversity metrics for each group. Table E. PERMANOVA outputs for the interaction between infection status and behavioral metrics on beta diversity metrics ( [file pntd.0013088.s001.docx]

**Supplementary Materials**

Does schistosome infection affect behavior through the gut-brain axis?

Leigh Combrink^1,†^, Johannie M. Spaan^2,†^, Alexis Perret^3^, Thomas Maehara^2^, Britney Hyun^2^, Dana Parker^2^, Jennifer L. Johns^4^, Michael S. Blouin^5^, Kathy Magnusson^4,6^, Michelle L. Steinauer^2^

^1^ School of Natural Resources and the Environment, University of Arizona, Tucson, AZ, United States

^2^ Department of Biomedical Sciences, Western University of Health Sciences, Lebanon, OR, United States

^3^ Ecole Nationale Veterinaire d'Alfort, Maisons-Alfort, France

^4^ Department of Biomedical Sciences, College of Veterinary Medicine, Oregon State University, Corvallis, OR, United States

^5^ Department of Integrative Biology, Oregon State University, Corvallis, OR, United States

^6^Linus Pauling Institute, Oregon State University, Corvallis, OR, United States

^†^ These authors contributed equally and share first authorship.

**Supplementary Table A.** Breakdown of sample sizes by cage, group, cercariae dose and treatment, and infection status (Yes/No). Abbreviations: PZQ = praziquantel.

| **Cercariae Dose + Treatment:** | | **50** | | **75** | | **100 + PZQ** | | **0** | **0 + PZQ** | **TOTAL** |
| --- | --- | --- | --- | --- | --- | --- | --- | --- | --- | --- |
| **Infection status:** | | **Yes** | **No** | **Yes** | **No** | **Yes** | **No** | **No** | **No** |  |
| **Group #:** | **Cage #:** |  |  |  |  |  |  |  |  |  |
| **Group 1** | Cage 2 | 4 |  |  |  |  |  |  |  |  |
|  | Cage 3 |  |  | 2 | 2 |  |  |  |  |  |
|  | Cage 4 |  |  |  |  | 4 |  |  |  |  |
|  | Cage 5 |  |  |  |  |  |  | 4 |  |  |
|  | Cage 6 |  |  |  |  |  |  |  | 4 |  |
|  | Cage 8 | 4 |  |  |  |  |  |  |  |  |
|  | Cage 9 |  |  | 4 |  |  |  |  |  |  |
|  | Cage 10 |  |  |  |  | 4 |  |  |  |  |
|  | Cage 11 |  |  |  |  |  |  | 4 |  |  |
|  | Cage 12 |  |  |  |  |  |  |  | 4 |  |
| **Total Group 1:** |  | **8** |  | **6** | **2** | **8** |  | **8** | **8** | **40** |
| **Group 2** | Cage 22 | 3 | 1 |  |  |  |  |  |  |  |
|  | Cage 23 |  |  | 3 | 1 |  |  |  |  |  |
|  | Cage 24 |  |  |  |  | 4 |  |  |  |  |
|  | Cage 25 |  |  |  |  |  |  | 4 |  |  |
|  | Cage 26 |  |  |  |  |  |  |  | 4 |  |
|  | Cage 28 | 3 |  |  |  |  |  |  |  |  |
|  | Cage 29 |  |  | 3 |  |  |  |  |  |  |
|  | Cage 30 |  |  |  |  | 3 |  |  |  |  |
|  | Cage 31 |  |  |  |  |  |  | 2 |  |  |
|  | Cage 32 |  |  |  |  |  |  |  | 3 |  |
| **Total Group 2:** |  | **6** | **1** | **6** | **1** | **7** |  | **6** | **7** | **34** |
| **Total (Group 1 + 2):** |  | **14** | **1** | **12** | **3** | **15** |  | **14** | **15** | **74** |


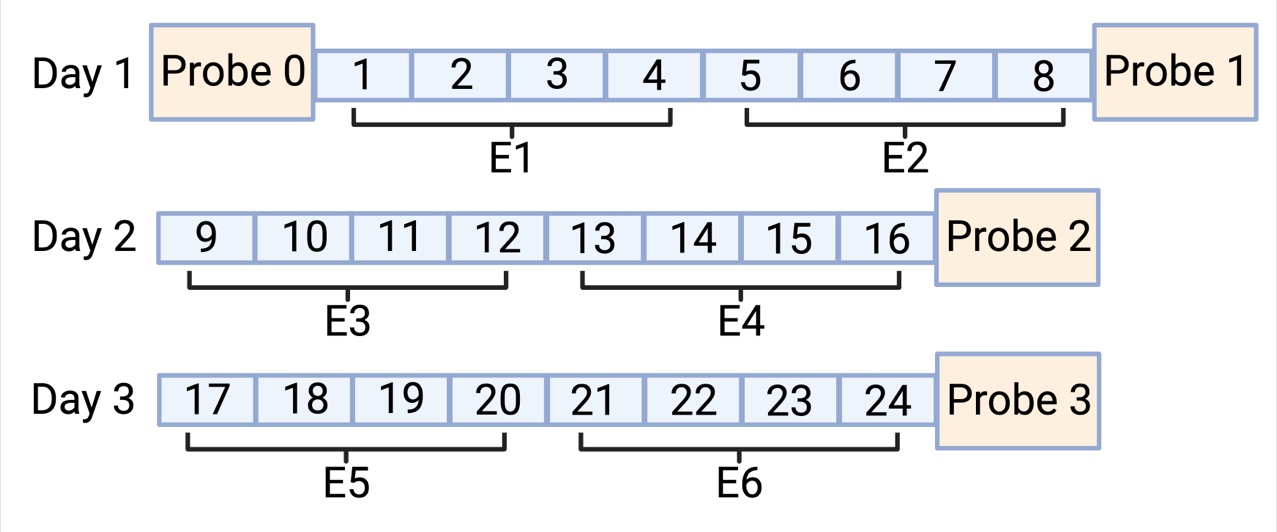


**Supplementary Figure A.** Diagram displaying the Morris Water Maze experiment over three days. Twenty-four place trials, indicated by blue boxes, were conducted and four probe trials were conducted. After an introduction period in which the mice swim to a visible platform, the experimental trials were conducted. During place trials, the platform is submerged, and the mouse is placed in a random entry point of the water tank. The mice use visual cues, shapes posted on four sides of the tank, to locate the platform. Swimming is tracked by an overhead camera and the distance, speed of swimming, and time to find the platform is recorded. The expectation is that the time to find the platform will decrease over time as the mouse learns the location of the platform based on the spatial cues (spatial learning). For statistical analysis, the average corrected cumulative distance for each mouse was calculated by taking the average of four consecutive place trials (E1-E6). For these trials, the platform is removed, and movements are tracked. See manuscript text for details of statistical analysis. Created in BioRender. Steinauer, M. (2025) https://BioRender.com/f81w865.

*Supporting data for why we analyzed microbiome group datasets independently—.*


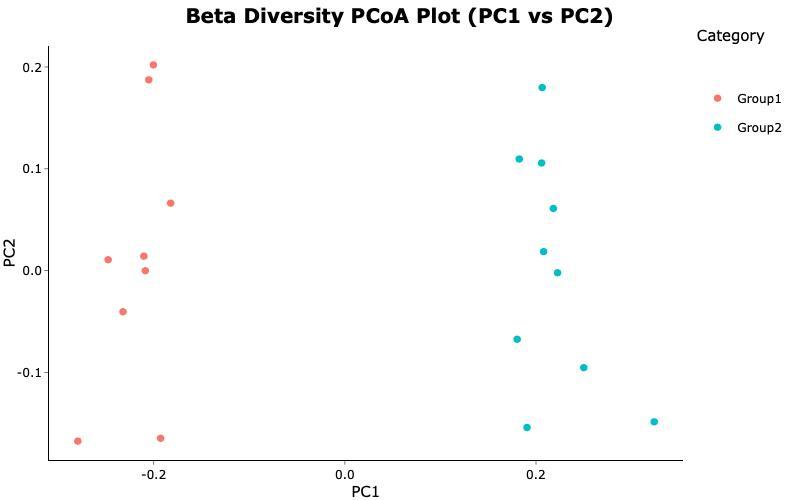


**Supplementary Figure B** A principal coordinate analysis (PCoA) plot created using the matrix of paired-wise distance between samples calculated by the Bray Curtis dissimilarity index. Each dot on the figure represents the whole microbial composition profile for each pooled sample across both groups. Samples with similar microbial composition profiles are closer to each other. Despite being sourced from the same laboratory and reared under similar conditions, and our attempts to homogenize gut microbiome composition between cohorts by housing group 2 mice in bedding collected from group 1’s cages, initial analysis of microbiome composition indicated that majority of the variance (PC1 axis 41.7%) was owing to group membership. This made it essential to analyze group datasets independently. Variation explained by PC2 and PC3 was 11.5% and 7.8%, respectively.

*Exclusion of praziquantel treated mice associated with 10 weeks post exposure fecal microbiome data—.*

**
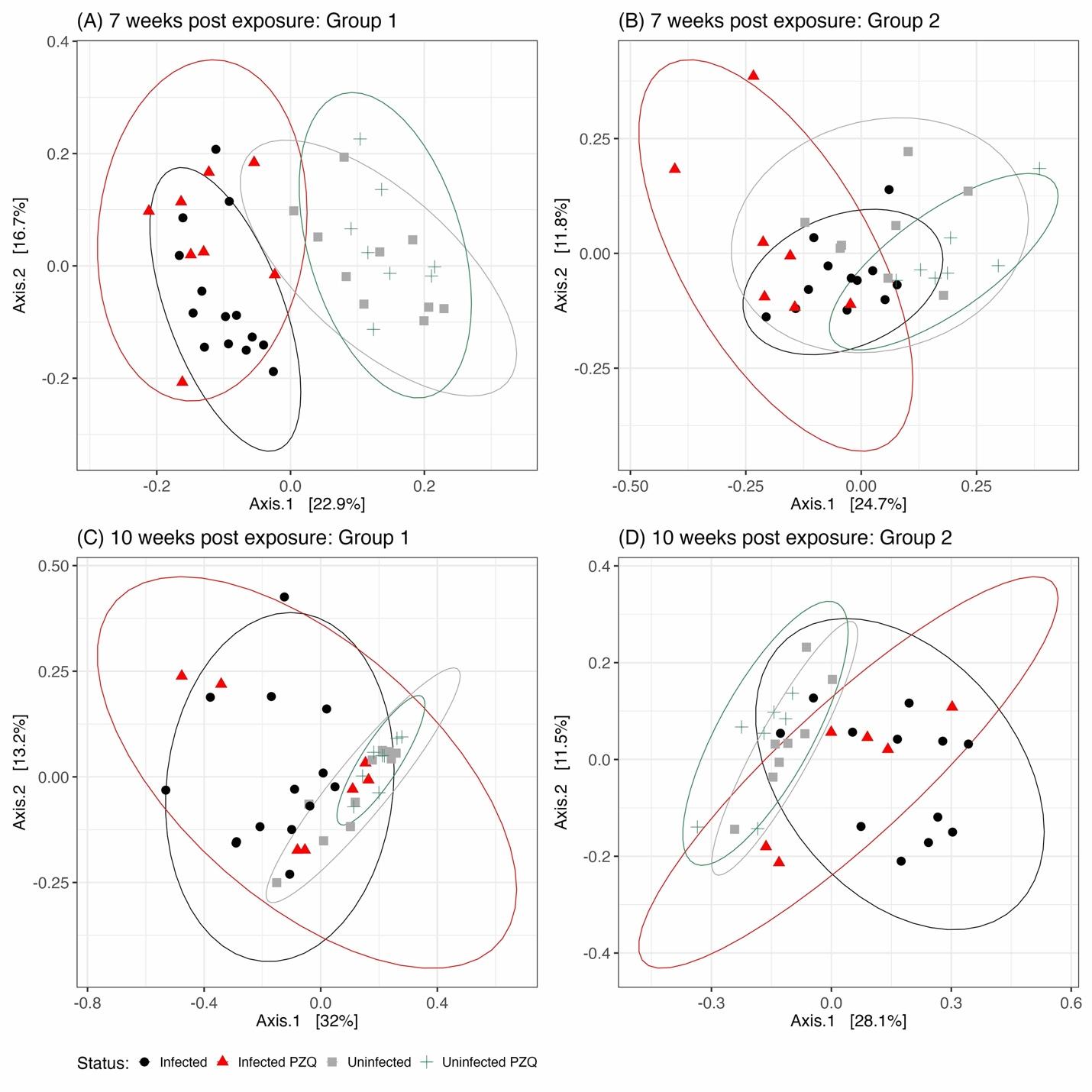
**

**Supplementary Figure C** Summary Principal coordinate analysis (PCoA) plots comparing infected, infected with praziquantel, control and control with praziquantel mice for (A) group 1, 7 weeks post exposure (n=40), (B) group 2, 7 weeks post exposure (n=34), (C) group 1, 10 weeks post exposure (n=40), and (D) group 2, 10 weeks post exposure (n=34). Full data for the 10 week time point, including the mice that received praziquantel treatment. The 7-week time point is separated by praziquantel treated mice even though the treatment were applied post fecal sample collection.


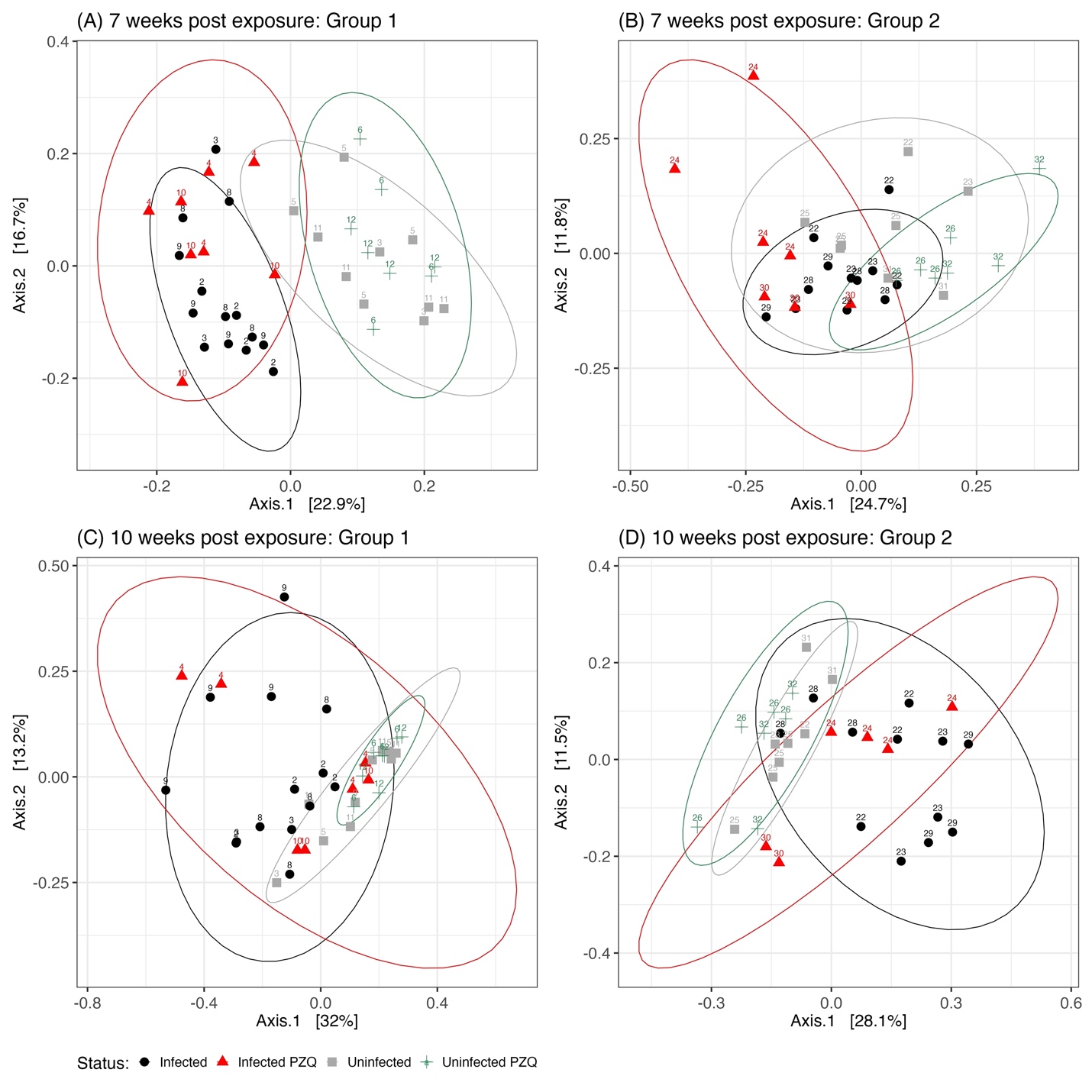


**Supplementary Figure D** Summary Principal coordinate analysis (PCoA) plots comparing infected, infected with praziquantel, control and control with praziquantel mice for (A) group 1, 7 weeks post exposure (n=40), (B) group 2, 7 weeks post exposure (n=34), (C) group 1, 10 weeks post exposure (n=40), and (D) group 2, 10 weeks post exposure (n=34). Full data for the 10 week time point, including the mice that received praziquantel treatment. The 7-week time point is separated by praziquantel treated mice even though the treatment were applied post fecal sample collection. The numbers indicate the cage number for all mice.

**
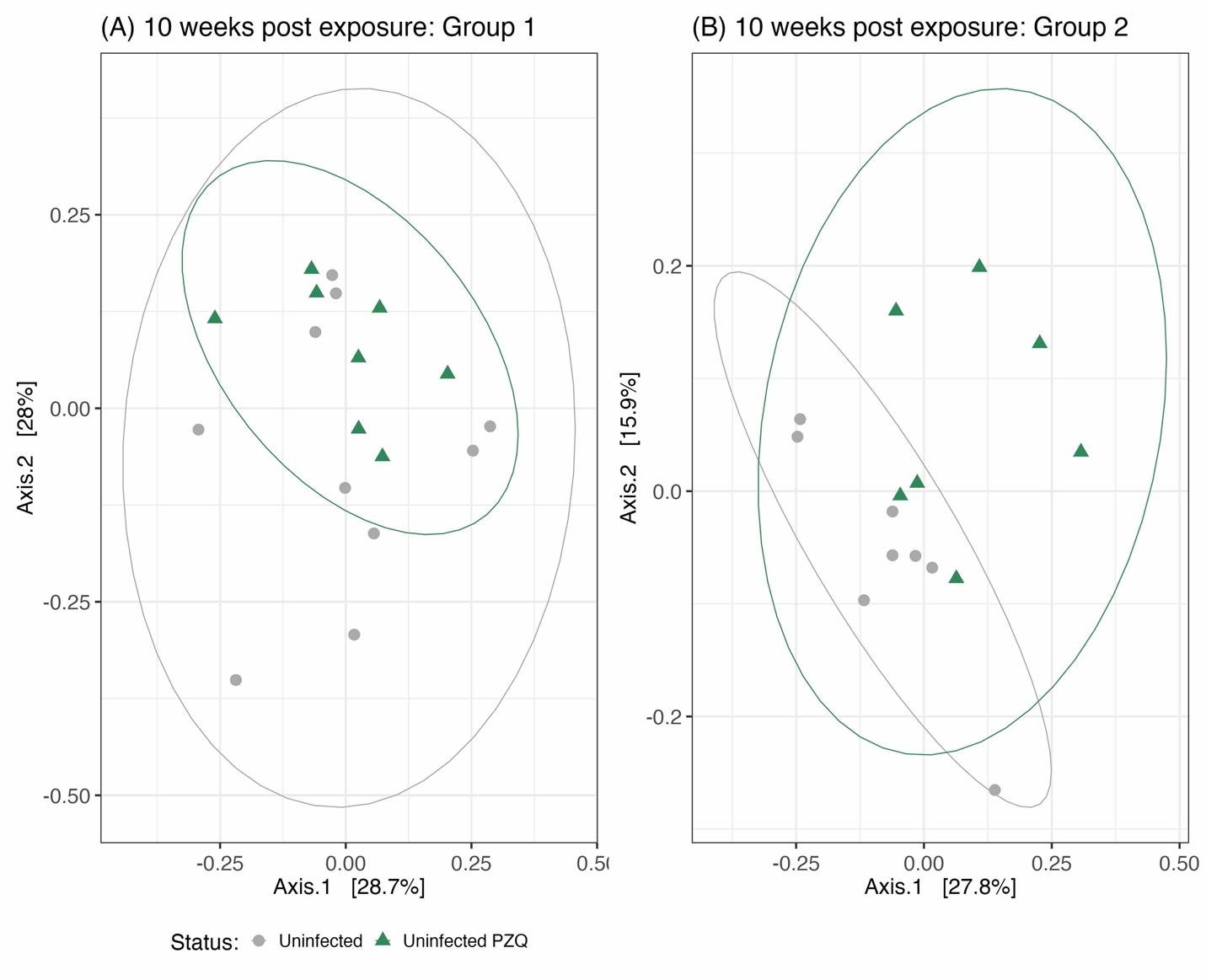
**

**Supplementary Figure E** Principal coordinate analysis (PCoA) plots comparing control mice (gray, circles) with praziquantel control mice (sea green, triangles) at 10 weeks post exposure for (A) group 1 (n=16) and (B) group 2 (n=13). Group 2 mice showed a praziquantel administration effect on species composition (PERMANOVA, *F*_1,14_ = 2.52, *P* = 0.0060), but group 1 mice did not (PERMANOVA, *F*_1,17_ = 1.54, *P* = 0.1518).

*For the behavioral data that matched with fecal microbiome data at 7 weeks post exposure—.*

**Supplementary Table B** Summary of Mann-Whitney U test outputs for comparisons between control mice and control praziquantel mice across all behavioral metrics, to ensure that our praziquantel (PZQ) administration did not significantly affect the mouse behavior outcomes. Abbreviations: OF = Open field; NO = Novel object; DI = Discrimination index.

|  | Control vs Control PZQ | |
| --- | --- | --- |
| Variables: | ***W*** | ***P*-value** |
| Marble burying (>50%) | 86.5 | 0.1998 |
| OF - % Permanence time spent in outer boundary (zone 1) | 102.5 | 0.5243 |
| OF - # Entries into the center (zone 2) | 117 | 0.9454 |
| OF - Latency time before entering zone 2 (the center) | 88.50 | 0.2333 |
| OF - Total distance traveled | 118 | 0.9844 |
| NO - DI Familiarization trial | 104.5 | 0.7665 |
| NO DI after 60 min retention time | 91.5 | 0.4054 |
| NO DI after 24 hours retention time | 104 | 0.5701 |

**Supplementary Table C** Summary output from Wilcoxon rank sum tests to determine the effect of infection status on alpha diversity metrics for each group and time comparison.

| Alpha diversity metrics: | **Group 1** | | **Group 2** | |
| --- | --- | --- | --- | --- |
|  | Week 7 | Week 10 | Week 7 | Week 10 |
|  | Infected vs.  Uninfected | Infected vs.  Uninfected | Infected vs.  Uninfected | Infected vs.  Uninfected |
| Species richness | *W* = 196, *P* = 0.9675 | *W* = 87, *P* = 0.3338 | *W* = 118.5, *P* = 0.4147 | *W* = 32.5, *P* = 0.2464 |
| Species evenness | *W* = 197, *P* = 0.9893 | *W* = 96, *P* = 0.1375 | *W* = 104, *P* = 0.1901 | *W* = 71, *P* = 0.0825 |
| Shannon index | *W* = 190, *P* = 0.8402 | *W* = 95, *P* = 0.1541 | *W* = 105, *P* = 0.2022 | *W* = 65, *P* = 0.2083 |


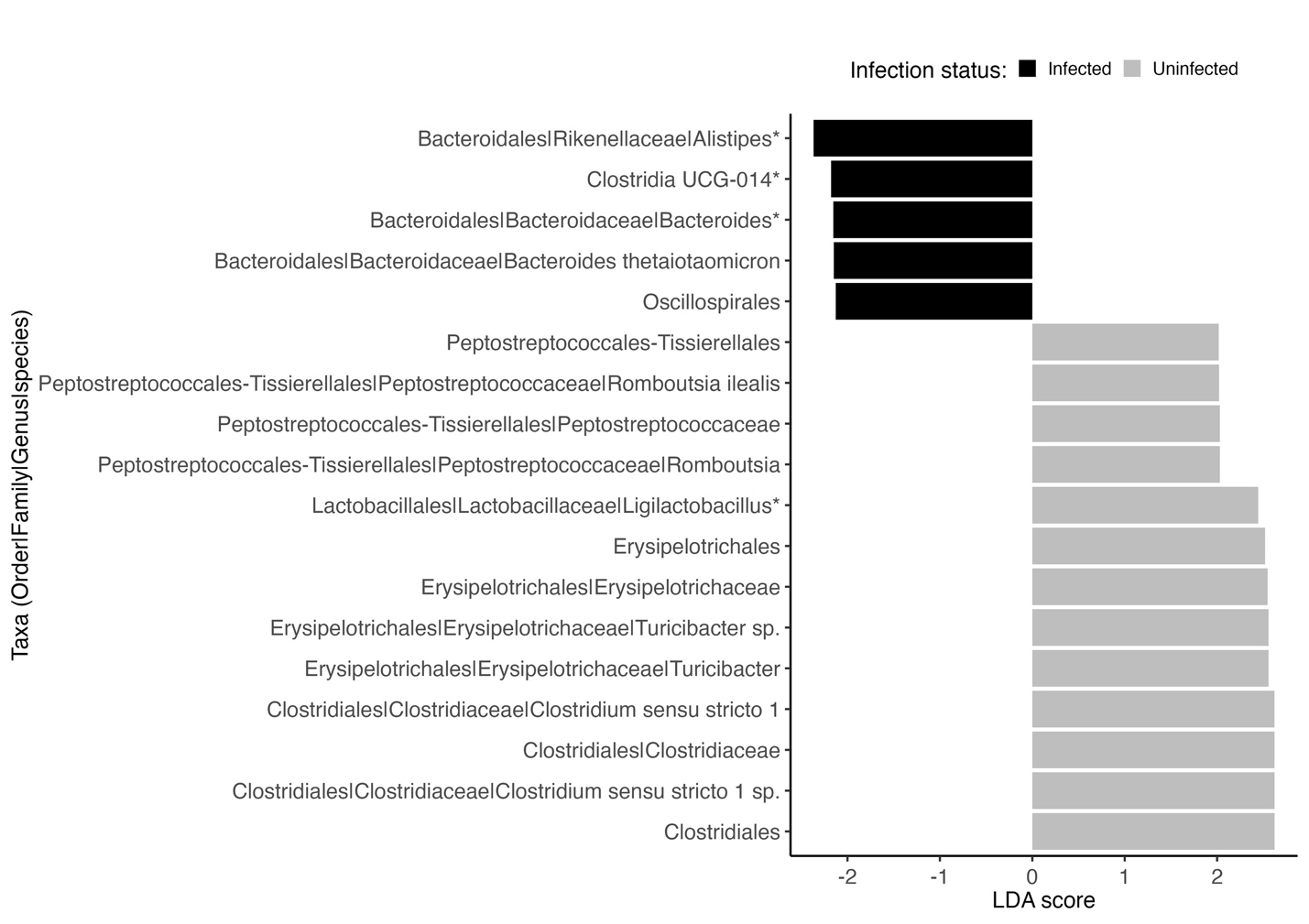


**Supplementary Figure F** Summary of the Linear Discriminant Analysis Effect Size (LEfSe) analysis comparing the taxa that differed significantly between schistosome infected (black bars, n=22) and uninfected (gray bars, n=18) mice from group 1 at 7 weeks post exposure. For simplicity, duplicate taxa with the exact same LDA scores and p-values were represented as a single entry and indicated by an asterisk.

**
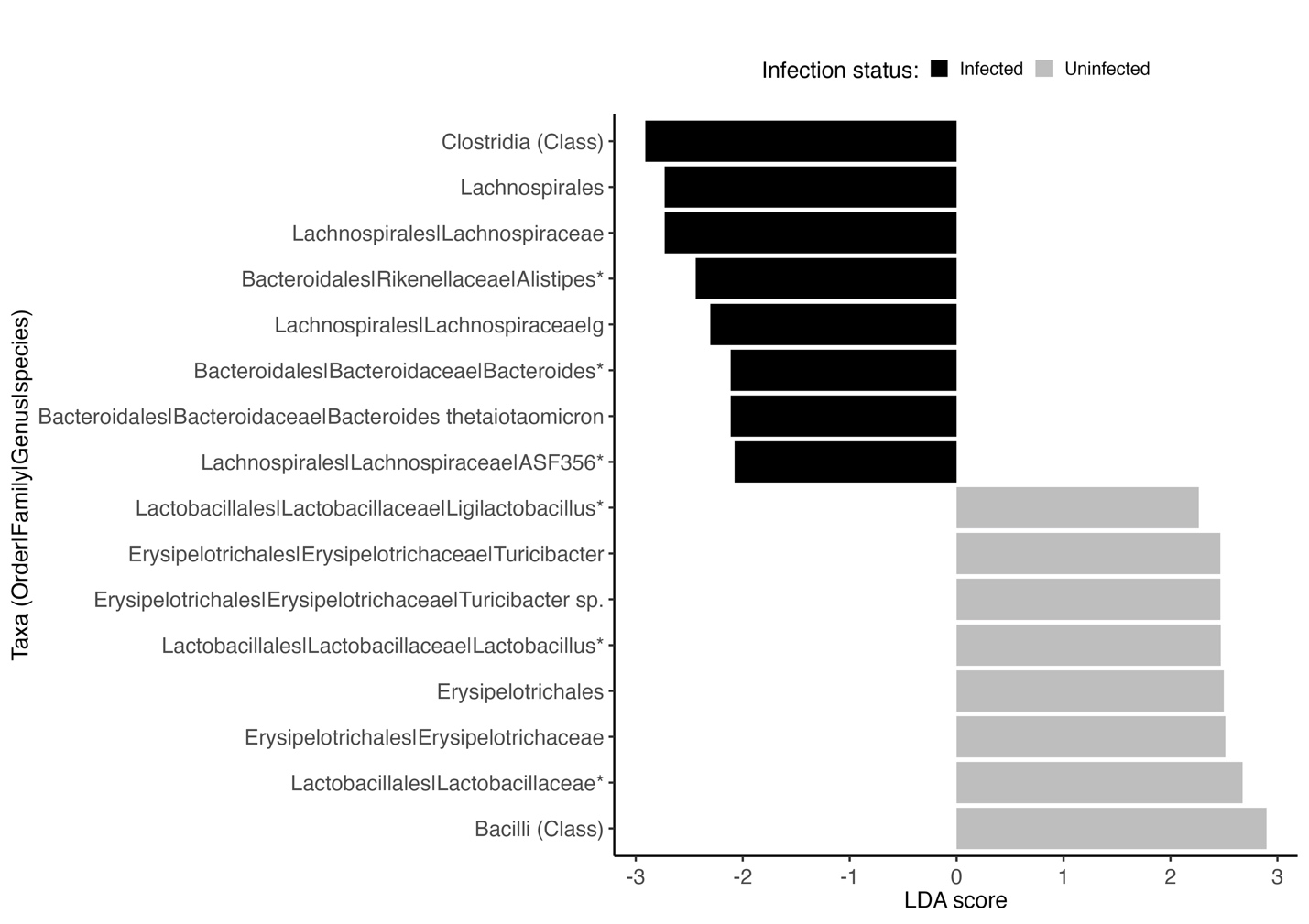
**

**Supplementary Figure G** Summary of the Linear Discriminant Analysis Effect Size (LEfSe) analysis comparing the taxa that differed significantly between schistosome infected (black bars, n=19) and uninfected (gray bars, n=15) mice from group 2 at 7 weeks post exposure. For simplicity, duplicate taxa with the exact same LDA scores and p-values were represented as a single entry and indicated by an asterisk.


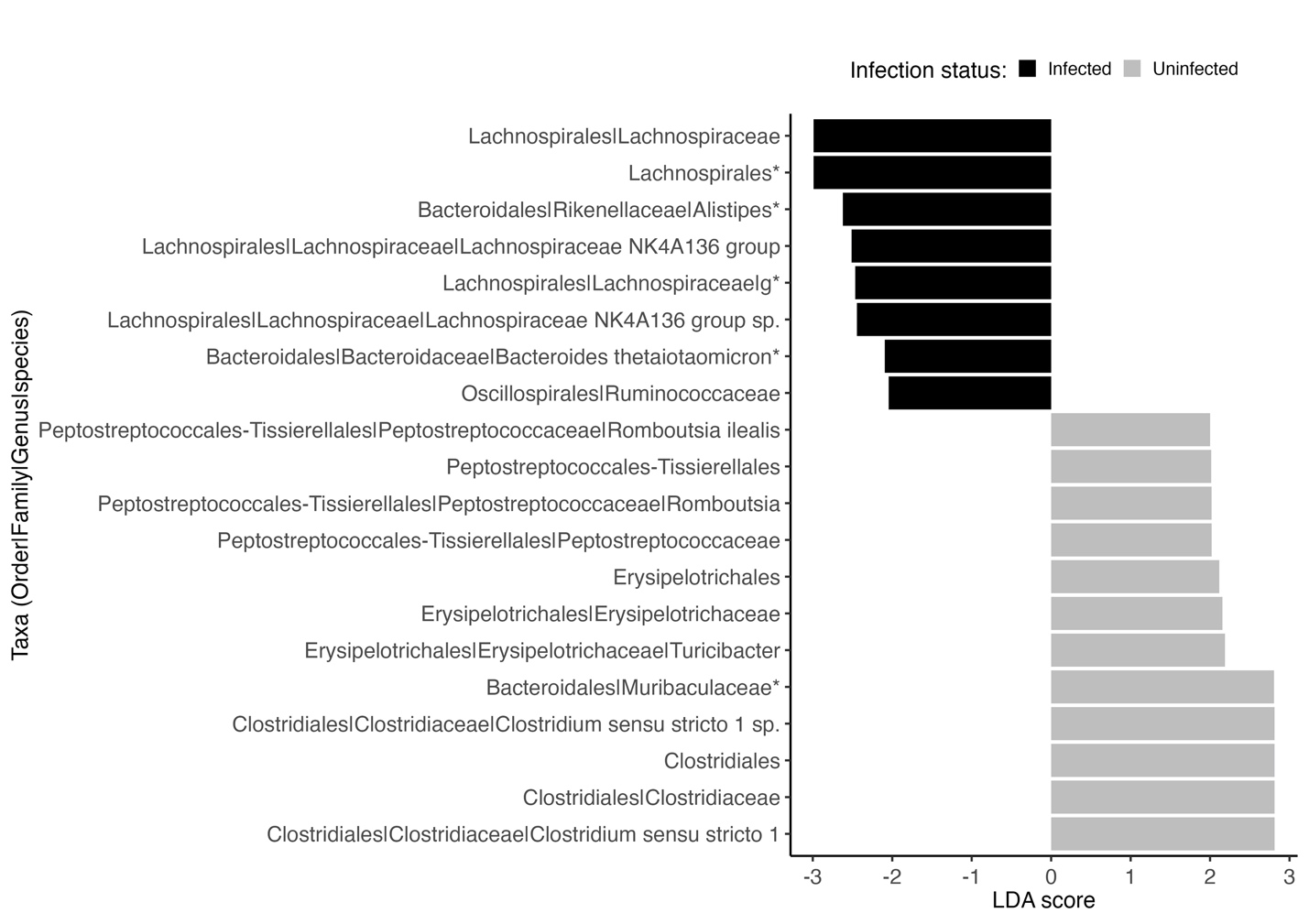


**Supplementary Figure H** Summary of the Linear Discriminant Analysis Effect Size (LEfSe) analysis comparing the taxa that differed significantly between schistosome infected (black bars, n=14) and uninfected (gray bars, n=10) mice from group 1 at 10 weeks post exposure. For simplicity, duplicate taxa with the exact same LDA scores and p-values were represented as a single entry and indicated by an asterisk.

**
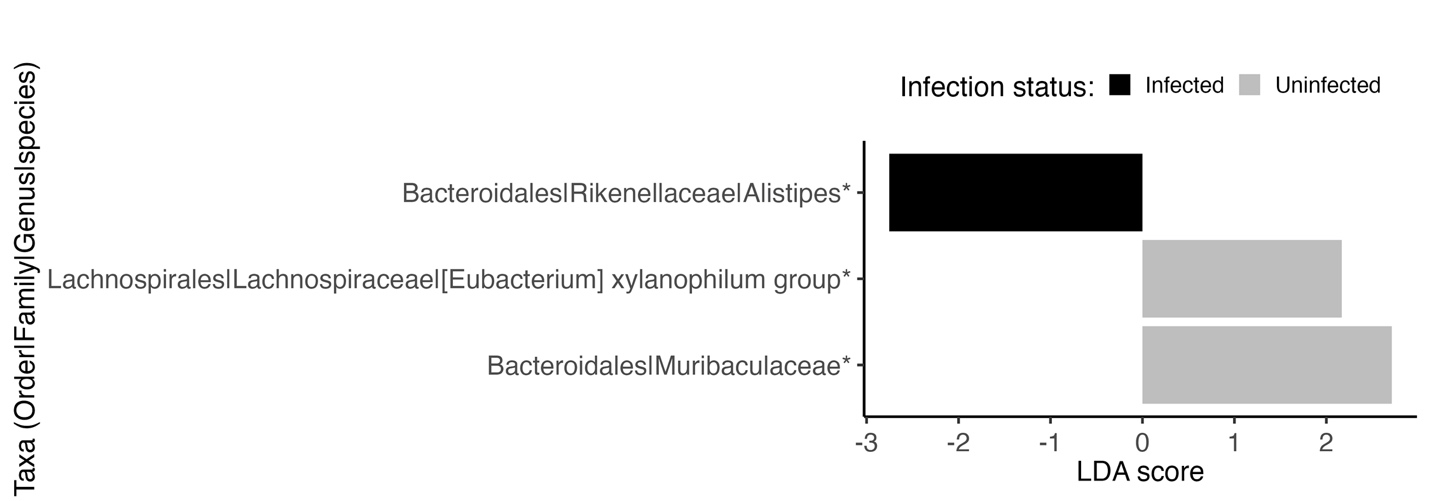
**

**Supplementary Figure I** Summary of the Linear Discriminant Analysis Effect Size (LEfSe) analysis comparing the taxa that differed significantly between schistosome infected (black bars, n=12) and uninfected (gray bars, n=8) mice from group 2 at 10 weeks post exposure. For simplicity, duplicate taxa with the exact same LDA scores and p-values were represented as a single entry and indicated by an asterisk.

**Supplementary Table D** Summary output from ANOVA with type III sum of squares from linear regression models (Group 1) or generalized linear models with negative binomial distribution (Group 2), to test for the interaction between infection status and behavioral metrics on alpha diversity metrics for each group. Abbreviations: OF = Open field; NO = Novel object; DI = Discrimination index, MWM = Morris ware maze.

| Behavioral metrics: | **Group 1** | | | **Group 2** | | |
| --- | --- | --- | --- | --- | --- | --- |
|  | Interaction between behavior and infection status | | | Interaction between behavior and infection status | | |
|  | Richness | Evenness | Shannon | Richness | Evenness | Shannon |
| OF - % Permanence time spent in outer boundary (zone 1) | *F*_1,39_ = 3.8, *P* = 0.0591 | *F*_1,39_ = 0.7, *P* = 0.4048 | *F*_1,39_ = 2.3, *P* = 0.1355 | *ꭓ^2^_df=1_* = 0.03, *P* = 0.8723 | *ꭓ^2^_df=1_* = 0.01, *P* = 0.9353 | *ꭓ^2^_df=1_* = 0.03, *P* = 0.8587 |
| OF - # Entries into the center (zone 2) | *F*_1,39_ < 0.01, *P* = 0.9880 | *F*_1,39_ = 5.5, ***P* = 0.0252** | *F*_1,39_ = 2.6, *P* = 0.1175 | *ꭓ^2^_df=1_* = 0.1, *P* = 0.7259 | *ꭓ^2^_df=1_* < 0.01, *P* = 0.9922 | *ꭓ^2^_df=1_* < 0.01, *P* = 0.9998 |
| OF - Latency time before entering zone 2 (the center) | *F*_1,39_ = 0.1, *P* = 0.7972 | *F*_1,39_ = 0.1, *P* = 0.7152 | *F*_1,39_ = 0.2, *P* = 0.6334 | *ꭓ^2^_df=1_* = 6.2, ***P* = 0.0129** | *ꭓ^2^_df=1_* = 0.01, *P* = 0.9107 | *ꭓ^2^_df=1_* = 0.1, *P* = 0.7088 |
| OF - Total distance traveled | *F*_1,39_ = 1.5, *P* = 0.2300 | *F*_1,39_ = 9.6, ***P* = 0.0037** | *F*_1,39_ = 7.7, ***P* = 0.0085** | *ꭓ^2^_df=1_* = 0.01, *P* = 0.9292 | *ꭓ^2^_df=1_* < 0.01, *P* = 0.9550 | *ꭓ^2^_df=1_* = 0.01, *P* = 0.9105 |
| NO - DI Familiarization trial | *F*_1,36_ = 0.1, *P* = 0.7185 | *F*_1,36_ = 0.1, *P* = 0.7662 | *F*_1,36_ = 0.2, *P* = 0.6804 | *ꭓ^2^_df=1_* = 0.1, *P* = 0.7743 | *ꭓ^2^_df=1_* < 0.01, *P* = 0.9978 | *ꭓ^2^_df=1_* < 0.01, *P* = 0.9713 |
| NO - DI after 60 min retention time | *F*_1,36_ = 0.1, *P* = 0.7251 | *F*_1,36_ = 1.3, *P* = 0.2597 | *F*_1,36_ = 1.1, *P* = 0.3022 | *ꭓ^2^_df=1_* = 3.6, *P* = 0.0582 | *ꭓ^2^_df=1_* < 0.01, *P* = 0.9969 | *ꭓ^2^_df=1_* = 0.01, *P* = 0.9099 |
| NO - DI after 24 hours retention time | *F*_1,36_ = 0.1, *P* = 0.7575 | *F*_1,36_ = 0.1, *P* = 0.7484 | *F*_1,36_ = 0.1, *P* = 0.7285 | *ꭓ^2^_df=1_* = 4.8, ***P* = 0.0286** | *ꭓ^2^_df=1_* < 0.01, *P* = 0.9856 | *ꭓ^2^_df=1_* = 0.01, *P* = 0.9411 |
| Marble burying (>50%) | *F*_1,39_ = 1.1, *P* = 0.2931 | *F*_1,39_ = 0.4, *P* = 0.5380 | *F*_1,39_ = 0.01, *P* = 0.9075 | *ꭓ^2^_df=1_* = 0.02, *P* = 0.8866 | *ꭓ^2^_df=1_* = 0.002, *P* = 0.9640 | *ꭓ^2^_df=1_* = 0.01, *P* = 0.9332 |
| MWM – Corrected cumulative distance place trial E1 | *F*_1,18_ = 1.7, *P* = 0.2091 | *F*_1,18_ = 3.2, *P* = 0.0917 | *F*_1,18_ = 3.4, *P* = 0.0861 | *F*_1,17_ = 0.4, *P* = 0.5269 | *F*_1,17_ = 0.7, *P* = 0.4315 | *F*_1,17_ = 1.8, *P* = 0.1993 |
| MWM – Corrected cumulative distance place trial E2 | *F*_1,18_ = 0.6, *P* = 0.4339 | *F*_1,18_ = 0.4, *P* = 0.5282 | *F*_1,18_ = 0.5, *P* = 0.4705 | *F*_1,17_ = 0.04, *P* = 0.8359 | *F*_1,17_ = 1.7, *P* = 0.2173 | *F*_1,17_ = 1.6, *P* = 0.2229 |
| MWM – Corrected cumulative distance place trial E3 | *F*_1,18_ = 0.5, *P* = 0.4726 | *F*_1,18_ = 3.2, *P* = 0.0934 | *F*_1,18_ = 2.2, *P* = 0.1563 | *F*_1,17_ = 0.1, *P* = 0.8016 | *F*_1,17_ = 1.1, *P* = 0.3158 | *F*_1,17_ = 0.9, *P* = 0.3605 |
| MWM – Corrected cumulative distance place trial E4 | *F*_1,18_ = 0.7, *P* = 0.4242 | *F*_1,18_ = 1.1, *P* = 0.3122 | *F*_1,18_ = 1.2, *P* = 0.2904 | *F*_1,17_ = 0.03, *P* = 0.8668 | *F*_1,17_ = 0.4, *P* = 0.5287 | *F*_1,17_ = 0.5, *P* = 0.4888 |
| MWM – Corrected cumulative distance place trial E5 | *F*_1,18_ = 0.5, *P* = 0.4921 | *F*_1,18_ = 0.1, *P* = 0.7437 | *F*_1,18_ = 0.2, *P* = 0.6269 | *F*_1,17_ = 0.2, *P* = 0.6287 | *F*_1,17_ = 0.01, *P* = 0.9339 | *F*_1,17_ = 0.2, *P* = 0.6352 |
| MWM – Corrected cumulative distance place trial E6 | *F*_1,18_ = 0.1, *P* = 0.7663 | *F*_1,18_ = 0.03, *P* = 0.8734 | *F*_1,18_ < 0.01, *P* = 0.9989 | *F*_1,17_ = 1.7, *P* = 0.2161 | *F*_1,17_ = 0.2, *P* = 0.6795 | *F*_1,17_ = 0.9, *P* = 0.3466 |
| MWM – Average corrected proximity from platform probe trial PR0 | *F*_1,18_ = 1.9, *P* = 0.1924 | *F*_1,18_ = 2.2, *P* = 0.1599 | *F*_1,18_ = 2.6, *P* = 0.1274 | *F*_1,17_ = 0.7, *P* = 0.4093 | *F*_1,17_ = 0.8, *P* = 0.3799 | *F*_1,17_ = 2.1, *P* = 0.1654 |
| MWM – Average corrected proximity from platform probe trial PR1 | *F*_1,18_ = 1.2, *P* = 0.2979 | *F*_1,18_ = 0.05, *P* = 0.8291 | *F*_1,18_ = 0.04, *P* = 0.8426 | *F*_1,17_ = 0.2, *P* = 0.6609 | *F*_1,17_ = 0.4, *P* = 0.5136 | *F*_1,17_ = 0.2, *P* = 0.6321 |
| MWM – Average corrected proximity from platform probe trial PR2 | *F*_1,18_ = 0.3, *P* = 0.5726 | *F*_1,18_ = 0.2, *P* = 0.6777 | *F*_1,18_ = 0.02, *P* = 0.9026 | *F*_1,17_ = 2.1, *P* = 0.1686 | *F*_1,17_ = 0.1, *P* = 0.7118 | *F*_1,17_ = 2.4, *P* = 0.1443 |
| MWM – Average corrected proximity from platform probe trial PR3 | *F*_1,18_ = 5.3, ***P* = 0.0361** | *F*_1,18_ = 2.0, *P* = 0.1760 | *F*_1,18_ = 3.5, *P* = 0.0793 | *F*_1,17_ = 0.1, *P* = 0.8222 | *F*_1,17_ = 1.3, *P* = 0.2686 | *F*_1,17_ = 1.4, *P* = 0.2598 |

**Notes:** Linear regression with Anova and type III sum of squares for group 1 week 7 of marble burying and open field metrics (due to alpha diversity metrics being normally distributed-Shapiro-Wilk normality test). Generalized linear model with negative binomial distribution due to non-normal alpha diversity metrics for group 2 week 7. Log transformation did not improve the fit. Again, linear regression with Anova and type III sum of squares for week 10 of Morris water maze (due to alpha diversity metrics being normally distributed-Shapiro-Wilk normality test).

**Supplementary Table E** Summary output from PERMANOVAs to test for the interaction between infection status and behavioral metrics on beta diversity metrics (Bray-Curtis dissimilarity index) for each group. Abbreviations: OF = Open field; NO = Novel object; DI = Discrimination index, MWM = Morris ware maze.

|  | **Group 1** | **Group 2** |
| --- | --- | --- |
|  | Interaction between behavior and infection status | Interaction between behavior and infection status |
| OF - % Permanence time spent in outer boundary (zone 1) | *F*_1,39_ = 1.1, *P* = 0.3087 | *F*_1,31_ = 1.1, *P* = 0.3097 |
| OF - # Entries into the center (zone 2) | *F*_1,39_ = 1.2, *P* = 0.2857 | *F*_1,31_ = 0.8, *P* = 0.6723 |
| OF - Latency time before entering zone 2 (the center) | *F*_1,39_ = 0.7, *P* = 0.7193 | *F*_1,31_ = 1.6, *P* = 0.0799 |
| OF - Total distance traveled | *F*_1,39_ = 1.4, *P* = 0.1788 | *F*_1,31_ = 0.7, *P* = 0.7662 |
| NO - DI Familiarization trial | *F*_1,36_ = 0.9, *P* = 0.5315 | *F*_1,30_ = 1.2, *P* = 0.2597 |
| NO - DI after 60 min retention time | *F*_1,36_ = 1.1, *P* = 0.3177 | *F*_1,30_ = 1.1, *P* = 0.3267 |
| NO DI after 24 hours retention time | *F*_1,36_ = 1.0, *P* = 0.4515 | *F*_1,30_ = 0.9, *P* = 0.4695 |
| Marble burying (>50%) | *F*_1,39_ = 1.4, *P* = 0.1618 | *F*_1,31_ = 1.6, *P* = 0.0569 |
| MWM – Corrected cumulative distance place trial E1 | *F*_1,18_ = 1.6, *P* = 0.1399 | *F*_1,17_ = 0.5, *P* = 0.9461 |
| MWM – Corrected cumulative distance place trial E2 | *F*_1,18_ = 2.8, ***P* = 0.0230** | *F*_1,17_ = 1.0, *P* = 0.4685 |
| MWM – Corrected cumulative distance place trial E3 | *F*_1,18_ = 2.1, *P* = 0.0539 | *F*_1,17_= 0.5, *P* = 0.9680 |
| MWM – Corrected cumulative distance place trial E4 | *F*_1,18_ = 0.5 *P* = 0.8432 | *F*_1,17_= 1.1, *P* = 0.3397 |
| MWM – Corrected cumulative distance place trial E5 | *F*_1,18_ = 1.4, *P* = 0.1678 | *F*_1,17_= 1.3, *P* = 0.2068 |
| MWM – Corrected cumulative distance place trial E6 | *F*_1,18_ = 1.8, *P* = 0.0899 | *F*_1,17_= 1.3, *P* = 0.1828 |
| MWM – Average corrected proximity from platform probe trial PR0 | *F*_1,18_ = 1.0, *P* = 0.4096 | *F*_1,17_= 0.6, *P* = 0.8981 |
| MWM – Average corrected proximity from platform probe trial PR1 | *F*_1,18_ = 0.6, *P* = 0.8502 | *F*_1,17_= 0.9, *P* = 0.5445 |
| MWM – Average corrected proximity from platform probe trial PR2 | *F*_1,18_ = 1.2, *P* = 0.2917 | *F*_1,17_= 1.0, *P* = 0.4555 |
| MWM – Average corrected proximity from platform probe trial PR3 | *F*_1,18_ = 1.9, *P* = 0.0609 | *F*_1,17_= 0.8, *P* = 0.6464 |
